# Integrated cerebro-splanchnic blood flow and regional oxygenation monitoring in transfused anemic preterm infants

**DOI:** 10.1038/s41598-026-53147-6

**Published:** 2026-06-23

**Authors:** Marwa Mohamed Farag, Hesham Abd El Rahim Ghazal, Ahmed Mohamed Said Abougabal, Asmaa Medhat Elsaudi

**Affiliations:** 1 Department of Pediatrics, Alexandria University Hospital, Alexandria, Egypt; 2 Radiodiagnosis and Interventional Radiology Department, Alexandria University, Alexandria, Egypt

**Keywords:** Diseases, Health care, Medical research, Physiology

## Abstract

The aim of this study was to assess the effect of packed red blood cell (PRBC) transfusion on blood flow velocity and near-infrared spectroscopy (NIRS)-measured regional oxygenation of both the brain and intestine in clinically stable anemic preterm infants with a gestational age of ≤ 32 weeks and a postnatal age of ≥ 3 weeks. This prospective observational study was conducted in the NICU of Alexandria University Maternity Hospital between March 2023 and December 2023. Clinically stable anemic preterm infants (n = 30) with hemoglobin < 9.5 g/dL and an indication for PRBC transfusion were included. Cerebral and mesenteric oxygenation were measured by NIRS before, during, and after transfusion. Superior vena cava flow (SVCF) and anterior cerebral artery (ACA) and celiac artery (CA) velocities were measured before and after transfusion. Comparative analyses were used to compare parameters before and after transfusion, and correlation tests were used to assess relationships between oxygenation and hemodynamic measures. SVCF, velocity–time integral (VTI), and SVC diameter decreased significantly after transfusion (p < 0.001). ACA peak systolic velocity (PSV), ACA end-diastolic velocity (EDV), and celiac artery PSV also decreased significantly after transfusion (p < 0.001). Splanchnic and cerebral oxygenation metrics increased significantly, whereas oxygen consumption and fractional tissue oxygen extraction decreased significantly after transfusion (p < 0.001). Delta changes in SVCF were correlated with splanchnic oxygenation metrics (p = 0.002–0.003), and heart rate was significantly correlated with both cerebral and splanchnic oxygenation metrics (p = 0.003–0.006). Cerebral and splanchnic oxygenation metrics, SpO2, SVC diameter, SVC VTI, SVCF, ACA PSV, ACA EDV, and celiac artery PSV changed significantly after blood transfusion in anemic preterm infants.

**Supplementary Information:**

The online version contains supplementary material available at 10.1038/s41598-026-53147-6.

## Introduction

Anemia of prematurity may impair oxygen delivery to organs, including the brain and intestine. Packed red blood cell (RBC) transfusion is a common, rapid, and effective intervention, particularly for preterm infants who are at risk of, or are experiencing, symptoms related to anemia and poor oxygenation^1^.

However, transfusion poses additional hazards for preterm infants beyond the complications seen at any age. Transfusing anemic preterm infants can be associated with periventricular leukomalacia, intraventricular hemorrhage, necrotizing enterocolitis, bronchopulmonary dysplasia, and retinopathy of prematurity^2^. This has made transfusion therapy in preterm infants a contentious topic in neonatal medicine.

Over the past two decades, researchers have focused on liberal versus restrictive transfusion guidelines in relation to survival and neurodevelopmental outcomes. These studies have provided data supporting restrictive transfusion thresholds based on the need for respiratory support, but hemodynamic and oxygenation responses and other clinical outcomes have been explored only to a limited extent^1,3,4^.

Monitoring tools such as jugular venous oxygen saturation, electroencephalography, event-related potentials, positron emission tomography, and functional magnetic resonance imaging can be used to assess cerebral oxygenation. NIRS is the only continuous, non-invasive bedside tool for monitoring both splanchnic and cerebral oxygen saturation, while Doppler imaging aids in repeated bedside evaluation of cerebral and intestinal hemodynamics in preterm infants^5–7^.

Understanding physiological responses to PRBC transfusion, including hematological, oxygenation, and hemodynamic changes, is important for tailoring management to the specific needs of each infant. Differences between symptomatic and asymptomatic infants may also provide insight into transfusion effectiveness and the potential need for additional therapeutic strategies.

This study aimed to evaluate the impact of PRBC transfusion on blood flow velocity and NIRS-measured regional oxygenation of both the brain and intestine in clinically stable anemic preterm infants (with no need for respiratory or inotropic support during the peri-transfusion period) with a gestational age of ≤ 32 weeks and a postnatal age of ≥ 3 weeks.

## Methods

### Study design and participants

This prospective study was conducted in the neonatal intensive care unit of Alexandria University Maternity Hospital between March 2023 and December 2023.

The study included 30 anemic preterm infants requiring PRBC transfusion, with gestational age ≤ 32 weeks and postnatal age ≥ 3 weeks. Twenty-four infants were symptomatic and six were asymptomatic, as outlined in the study flow chart (Supplementary Fig. 1).

At assessment, all enrolled infants had a closed patent ductus arteriosus, did not require continuous supplemental oxygen, were hemodynamically stable without inotropic infusion, and were free from sepsis and hemolysis. Infants with sepsis, hemolysis, congenital anomalies, or cardiorespiratory support were excluded.

### Transfusion protocol

All infants received PRBC transfusions according to guidelines for premature infants^1,8^. Each infant received 15 mL/kg of cross-matched PRBCs over 4 h using a syringe pump.

Symptomatic anemic infants (n = 24) were transfused if hemoglobin was < 9.5 g/dL and/or hematocrit was < 27%. Asymptomatic anemic infants (n = 6) were transfused if hemoglobin was < 7.5 g/dL and/or hematocrit was < 22%. Reported symptoms included tachycardia, failure to thrive, apnea, and desaturations.

### Clinical monitoring

Hemodynamic parameters, including heart rate, oxygen saturation, and blood pressure, were recorded 30 min before and after RBC transfusion. Heart rate and SpO2 were monitored using a Nellcor pulse oximeter (Medtronic, Minneapolis, MN, USA), and values were recorded only when a stable plethysmographic waveform was observed while the infant was calm and non-agitated.

Non-invasive systolic, diastolic, and mean blood pressure was measured using an oscillometric device with an appropriately sized cuff on the right upper limb. Measurements were obtained while the infant was calm and off-feeding.

Bronchopulmonary dysplasia was defined as the need for additional oxygen at 28 days of postnatal age according to NIH/Bancalari criteria. NEC was categorized using Bell’s criteria, and ROP was assessed according to the International Classification of Retinopathy of Prematurity^9–11^.

### Imaging and feeding status

To eliminate the confounding effects of postprandial hyperperfusion on splanchnic hemodynamics, all Doppler measurements were performed in a standardized fasting state. According to the institutional protocol for RBC transfusion in preterm infants, enteral feeds were withheld for 2 h before, during, and 2 h after transfusion. At the time of the study, all infants were receiving full enteral feeds (180 mL/kg/day) before the peri-transfusion period.

Echocardiographic and Doppler studies were performed using a GE Vivid iq premium machine (WUXI, China) with M-mode, 2D, color Doppler, and pulsed-wave Doppler capabilities. During scanning, infants were asleep or quietly resting in the supine position on a flat surface.

### Superior vena cava flow measurements

SVC diameter was assessed using a GE 12S-RS probe (5–11 MHz) through the parasternal view, with the beam angled to the right of the ascending aorta. Minimal and maximal diameters were averaged over three cardiac cycles using M-mode.

SVC velocity–time integral was measured from a low subcostal view using pulsed-wave Doppler. Mean velocity was averaged over five consecutive cardiac cycles.

SVCF was calculated as SVCF = (SVC VTI × (π × (mean SVC diameter^2 / 4) × heart rate) / body weight), and expressed as mL/kg/min^12^.

### Cerebral and splanchnic blood flow velocities

Anterior cerebral artery and celiac artery velocities were measured using a GE 8C-RS probe (3.5–10 MHz). The ultrasound software was used to analyze pulsed-wave Doppler waveforms, including peak systolic velocity, maximal end-diastolic velocity, and resistive index. Values were averaged over three consecutive cycles.

### Near-infrared spectroscopy

Cerebral (CrSO2) and splanchnic (SrSO2) oxygenation were recorded using an INVOS 5100C Cerebral/Somatic Oximeter Monitor (Medtronic) before transfusion, during transfusion, and 12–24 h after transfusion.

A neonatal brain sensor was applied to the left frontoparietal area and the second sensor was placed over the hypogastrium in the midline above the symphysis pubis.

NIRS measurements before and after transfusion were averaged over 30 min, while measurements during transfusion were averaged over 2 h beginning 30 min after transfusion commenced. At the same time, preductal SpO2 was measured using a pulse oximetry sensor applied to the right wrist.

Fractional tissue oxygen extraction was calculated as FTOE = (SpO2—rSO2) / SpO2. SFTOE and CFTOE refer to splanchnic and cerebral FTOE, respectively. The difference between SpO2 and rSO2 reflects oxygen extraction and oxygen consumption^13–15^.

### Sample size

A minimum sample size of 16 preterm infants with gestational age ≤ 32 weeks was calculated to detect the effect of RBC transfusion on cerebral flow velocity and a difference of 0.01 in resistive index with 80% power using a one-sample t-test at a significance level of 5%^16^. Sample size was calculated using NCSS 2004 and PASS 2000.

### Statistical analysis

Data were entered and analyzed using IBM SPSS Statistics version 20.0. The Kolmogorov–Smirnov test was used to assess normality.

Qualitative data were described using number and percentage. Quantitative data were described using range, mean, standard deviation, median, and interquartile range, as appropriate.

Repeated-measures ANOVA, Friedman test, paired t-test, and Wilcoxon signed-rank test were used to compare measures before, during, and after transfusion. The Mann–Whitney test was used to compare symptomatic and asymptomatic infants. Pearson correlation coefficients were used for normally distributed quantitative variables. Significance was set at 5%.

For pairwise comparisons, Bonferroni correction was applied by dividing 0.05 by the number of groups (0.0167).

## Results

This prospective observational study evaluated the impact of PRBC transfusion on cardiac, cerebral, and splanchnic blood flow and oxygenation in anemic preterm infants. Infants were assessed 30–60 min before and 12–24 h after PRBC transfusion, with specific measurements of cerebral and splanchnic blood flow velocities and SVCF.

The study included 30 preterm neonates who met the eligibility criteria. Of these, 24 were symptomatic, with tachycardia (n = 11), apnea (n = 4), desaturation attacks (n = 14), and failure to thrive (n = 2).

The median gestational age was 30 weeks (range, 29–32 weeks). The mean birth weight was 1.162 kg (range, 0.85–1.7 kg). The median number of PRBC transfusions was 3 (range, 1–6). Maternal risk factors, resuscitation needs, and perinatal course are presented in Supplementary Table 1a–d.

Supplementary Table 2a–c provide a full comparison between symptomatic and asymptomatic infants with respect to hematological profile, clinical and imaging hemodynamic parameters, and cerebro-splanchnic regional oxygenation measures. Hemoglobin and hematocrit were significantly lower in asymptomatic patients. SVC flow was relatively higher before transfusion than after transfusion in both groups, and the symptomatic group showed a greater post-transfusion drop in SVC flow.

**Fig. 1:**
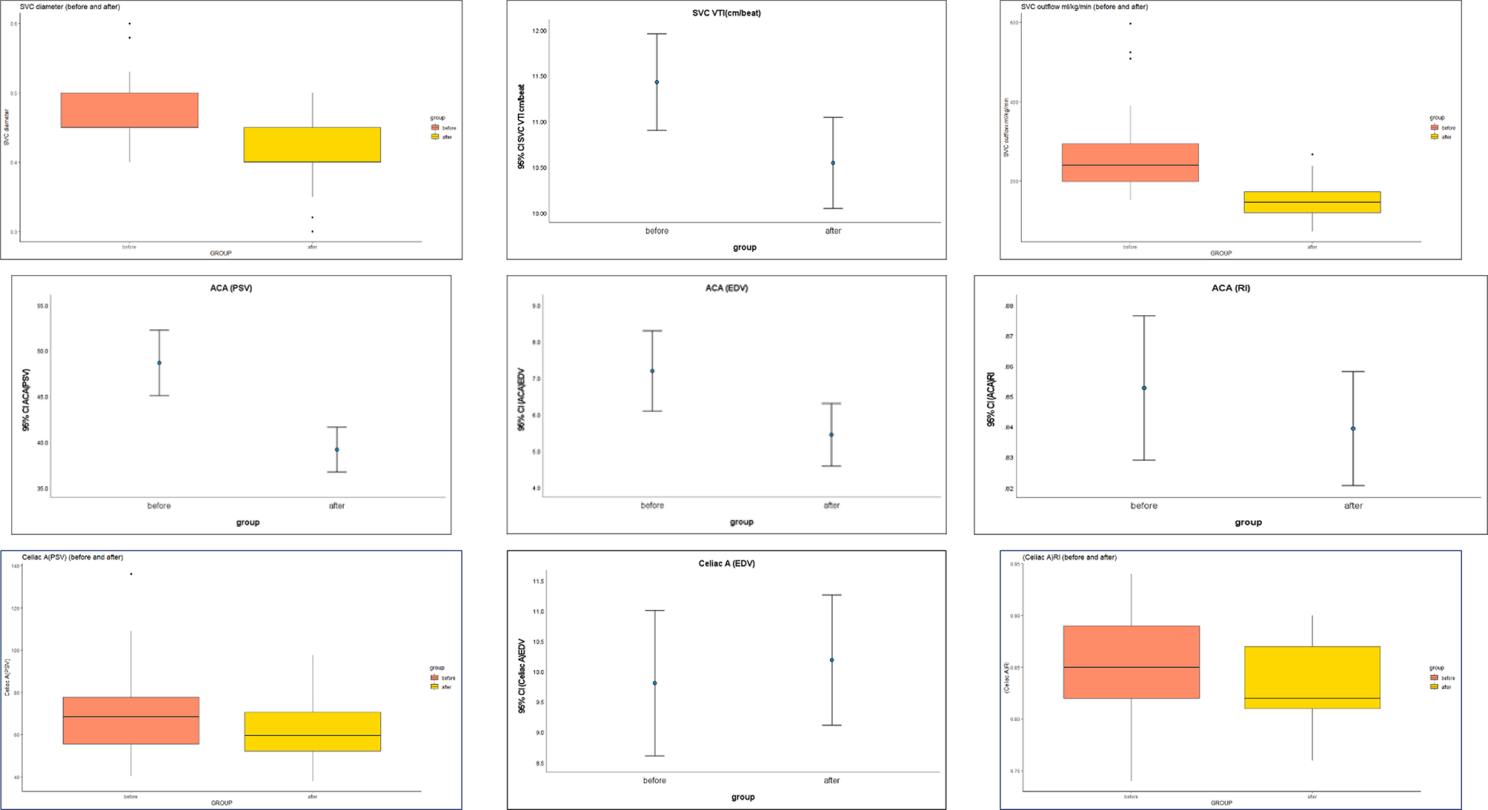
SVC diameter, VTI and flow are demonstrated in the upper panel before and after transfusion. ACA velocities (including PSV, EDV and RI) decrease after transfusion were demonstrated in the middle panel. For celiac artery velocities, EDV and RI are almost similar before and after transfusion. Albeit, Celiac artery PSV is higher before than after transfusion. SVC, superior vena cava; VTI, velocity time integral; PSV, peak systolic velocity; EDV, end diastolic velocity; RI, resistive index.

Table 1 summarizes hematological, weight, and clinical and imaging hemodynamic parameters before and after transfusion. Red blood cell indices and weight increased significantly after transfusion, whereas heart rate decreased significantly. SVCF, SVC VTI, and SVC diameter also decreased significantly after transfusion. ACA PSV, ACA EDV, and celiac artery PSV significantly decreased after transfusion. Echocardiographic and Doppler measured arterial flow velocimetry before and after transfusion are demonstrated in Fig. 1.

**Table 1:** Comparative analysis of hematological and hemodynamic parameters before and after blood transfusion.

Variable	Before	After	Statistical test value	P value
HR (beat/min)	167.5 (160–170)	140 (133–150)	1	0.001 b
Systolic BP (mmHg)	68.17 (5.58, 59–78)	68.97 (5.5, 57–79)	0.442	0.661 a
Diastolic BP (mmHg)	40.87 (5.22, 30–55)	41.07 (4.52, 32–53)	0.147	0.883 a
Mean BP (mmHg)	48 (45–51)	48 (45–51)	0.603	0.54 b
Hb (g/dL)	7.74 (0.59, 6.7–9.4)	10.11 (0.69, 8.8–11.8)	24.55	0.007 a
HCT (%)	22.65 (21.33–23.1)	29.15 (27.1–30.73)	4.78	0.002 b
MCV (fL)	87.6 (83.63–)	89.5 (86–93.5)	3.64	0.003 b
MCH (pg)	32 (31–33.35)	33.15 (32.23–34)	3.79	0.001 b
WBCs (× 10^3 cells/µL)	10.15 (8.4–13.5)	11.8 (9.33–12.85)	1.52	0.127 b
Platelet (× 10^3 cells/µL)	414.2 (135.8, 172–704)	389.87 (119.92, 216–659)	1.59	0.121 a
Weight (kg)	1.33 (0.22, 0.85–1.77)	1.36 (0.22, 0.88–1.8)	9.37	0.003 a
SVC diameter (cm)	0.45 (0.45–0.5)	0.4 (0.4–0.45)	4.18	0.001 b
SVC VTI (cm/beat)	11.43 (1.42, 8.3–13.6)	10.54 (1.33, 8.2–13)	3.36	0.001 b
SVCF (mL/kg/min)	239.8 (199.15–294.77)	147.15 (120.75–173.43)	4.78	0.004 b
ACA PSV (cm/s)	48.64 (9.59, 27–65)	39.14 (6.58, 26.3–54)	6.84	0.002 a
ACA EDV (cm/s)	7.18 (2.95, 2–13.7)	5.43 (2.3, 2.3–11.5)	4.7	0.001 a
ACA RI	0.85 (0.06, 0.75–1.08)	0.84 (0.05, 0.74–0.95)	1.24	0.224 a
CA PSV (cm/s)	68.35 (55.5–77.75)	59.5 (52.22–70.72)	3.84	0.001 b
Celiac A EDV (cm/s)	9.8 (3.21, 4–19.3)	10.18 (2.87, 5.2–16)	0.58	0.561 a
Celiac A RI	0.85 (0.82–0.89)	0.82 (0.81–0.87)	1.64	0.100 b

* p < 0.05 was considered statistically significant. Paired t-test (a) and Wilcoxon signed-rank test (b) were used as appropriate. Abbreviations: WBCs, white blood cells; SVC, superior vena cava; VTI, velocity time integral; SVCF, superior vena cava flow; ACA, anterior cerebral artery; PSV, peak systolic velocity; EDV, end-diastolic velocity; RI, resistive index; CA, celiac artery. Mean (SD, Range), Median (IQR).

**Fig. 2:**
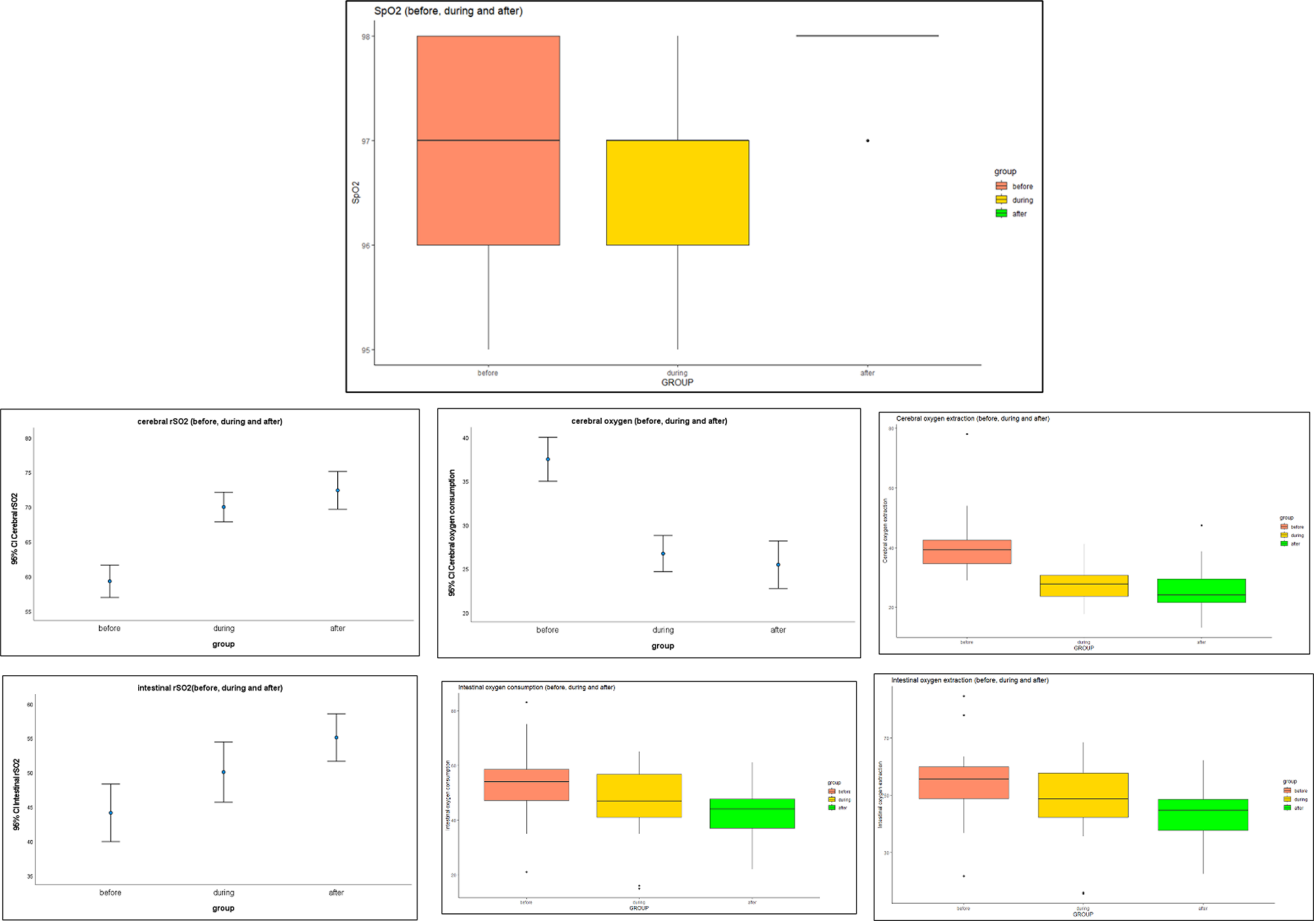
The SpO2 markedly improved after transfusion in relation to before and during transfusion. The cerebral oxygenation metrics (CrSO2, SpO2-CrSO2, CFTOE) before, during and after transfusion are demonstrated in the middle panel. The Splanchnic oxygenation metrics (SrSO2, SpO2-SrSO2, SFTOE) before, during and after transfusion are demonstrated in the lower panel. SrSO2, splanchnic regional oxygenation; CrSO2, cerebral regional oxygenation; FTOE, fractional tissue extraction of oxygen.

Table 2 and Fig. 2 summarize SpO2 and splanchnic and cerebral oxygenation metrics before, during, and after transfusion. These parameters showed significant changes across the three time points.

**Table 2:** Comparative analysis of oxygenation parameters before, during, and after PRBC transfusion.

Variable	Before	During	After	Pairwise p values	Test value	Overall p value
SpO2 (%)	97 (96–98)	97 (96–98)	98 (97–98)	B-D 0.74; B-A < 0.001; A-D < 0.001	29.83	< 0.001 b
CrSO2 (%)	59.27 (6.25, 45–73)	69.97 (5.71, 57–80)	72.37 (7.29, 51–85)	B-D < 0.001; B-A < 0.001; A-D < 0.001	53.16	< 0.001 a
SpO2-CrSO2 (%)	37.47 (6.73, 23–53)	26.7 (5.53, 17–40)	25.43 (7.26, 13–46)	B-D < 0.001; B-A < 0.001; A-D 0.101	48.06	< 0.001 a
CFTOE	39.2 (34.7–42.58)	27.8 (23.73–30.73)	24.05 (21.65–29.5 )	B-D 0.003; B-A 0.008; A-D 0.48	38.23	< 0.001 b
SrSO2 (%)	44.1 (11.23, 15–75)	50.03 (11.75, 30–80)	55.07 (9.25, 37–75)	B-D 0.002; B-A < 0.001; A-D 0.005	21.93	< 0.001 a
SpO2-SrSO2 (%)	54 (47.25–58.50)	47 (41–56.75)	44 (37–47.75)	B-D 0.004; B-A < 0.001; A-D 0.03	18.5	< 0.001 b
SFTOE	55.6 (48.9–60)	48.7 (42.33–57.75)	44.8 (37.7–48.65)	B-D 0.004; B-A 0.003; A-D 0.017	19.5	< 0.001 b

* Overall p < 0.05 was considered statistically significant. Repeated-measures ANOVA (a), Friedman test (b), paired t-test (c), and Wilcoxon signed-rank test (d) were used as appropriate. Bonferroni-corrected significance threshold for pairwise comparison: 0.0167. SpO2, peripheral oxygen saturation; CrSO2, cerebral regional oxygenation; SrSO2, splanchnic regional oxygen saturation; SFTOE, splanchnic fractional tissue oxygen extraction; CFTOE, cerebral fractional tissue oxygen extraction. Median (IQR), Mean(SD, range).

Supplementary Table 3a and b demonstrate correlations between hemodynamic and oxygenation parameters before and after transfusion. Before transfusion, SVC diameter, VTI, and flow, as well as ACA RI, were correlated with splanchnic oxygenation metrics. After transfusion, SVC VTI and celiac artery EDV were correlated with cerebral oxygenation measures. Supplementary Table 3c shows that delta changes in SVCF were correlated with splanchnic oxygenation metrics and that heart rate was significantly correlated with both cerebral and splanchnic oxygenation metrics.

Representative echocardiographic and Doppler images of SVC measurements, ACA and celiac artery velocimetry, and NIRS-measured cerebro-splanchnic oxygenation are presented in Supplementary Figs. 2–11. Supplementary Table 4a–c present hemodynamic and NIRS-measured regional oxygenation parameters in relation to BPD, NEC, and ROP.

## Discussion

To our knowledge, this is the first study to integrate both splanchnic and cerebral hemodynamics and oxygenation metrics, together with SVCF measures, to assess the impact of blood transfusion on anemic preterm infants. It is also among the few studies to explore the effect of transfusion on SVCF^16^.

Hemodynamically significant anemia can be defined on the basis of left ventricular output, stroke volume, heart rate, and ACA PSV. These parameters vary before and after transfusion and between anemic and non-anemic infants^17^.

Anemia leads to increased cardiac output to enhance oxygen delivery to tissues, resulting in elevated PSV, EDV, and SVC measurements including flow, diameter, and VTI. These measures decreased significantly after transfusion, indicating improved cerebral oxygenation and reduced cardiac output demand. A significant reduction in ACA EDV and ACA PSV together with stable systolic and diastolic blood pressure reflects nearly mature cerebral autoregulation in these infants. However, there were no changes in the resistive indices of the celiac and anterior cerebral arteries.

Chetan et al. observed a notable reduction in PSV after transfusion^18^, while Dani et al. found a decrease in EDV and an increase in RI, suggesting that transfusion enhanced cerebral oxygen supply and likely increased cerebral vascular resistance^19^. Krimmel et al. demonstrated that the normal postprandial increase in mesenteric blood flow velocity is blunted following transfusion^20^. Splanchnic circulation acts as a major blood reservoir, and celiac artery flow has been validated as a reliable proxy for regional perfusion changes^21,22^.

SVCF reflects both cardiac output and cerebral blood flow. It is estimated that 80% of total SVCF is venous return from the head, although exact ranges have not been established^23–26^. The use of SVCF in monitoring preterm infants is increasing in neonatal intensive care units.

Our study demonstrated significant decreases in SVC diameter, VTI, and flow after transfusion. Similar findings were reported by Banerjee et al., who found that PRBC transfusion resulted in significant increases in blood pressure and improvements in cerebral oxygenation indices, with significant decreases in ACA time-averaged mean velocity and SVCF after transfusion^16^.

NIRS-derived CrSO2 and SrSO2 represent mixed tissue saturation values and allow assessment of the balance between oxygen supply and consumption in the brain and intestine^27,28^. In the present study, CrSO2 increased significantly throughout the transfusion process, and both cerebral and splanchnic oxygen extraction metrics decreased significantly.

Measurements during transfusion were obtained 30 min after the start of transfusion and continued for 2 h, whereas the total transfusion lasted 4 h. This suggests that pronounced improvement in oxygenation occurred after approximately half of the transfused volume had been administered, corresponding to about 8 mL/kg of PRBCs. This observation may be relevant when considering smaller transfusion volumes in anemic neonates.

These findings are consistent with Chen et al., who found that both 20 mL/kg and 15 mL/kg PRBC transfusions significantly increased CrSO2 and SrSO2 and decreased FTOE in anemic preterm infants^29^. Similarly, Van Hoften et al. showed that lower pre-transfusion hemoglobin levels were strongly associated with reduced CrSO2 and increased CFTOE, and that transfusion improved cerebral oxygenation within 24 h^30^.

Bailey et al. also reported significant increases in both CrSO2 and SrSO2 following transfusion in symptomatic anemic preterm neonates, consistent with the present findings^31^. In addition, they found no correlation between hemoglobin and cerebro-splanchnic oxygenation metrics, similar to the current study.

White et al. reached a different conclusion, observing no significant changes in SrSO2, SFTOE, or SpO2-SrSO2 after transfusion^32^. Some researchers have linked regional oxygenation to clinical outcomes, with splanchnic oxygenation associated with NEC and cerebral saturation associated with neurodevelopmental outcomes. Chock et al. linked pre-transfusion cerebral saturation below 50% with long-term neurodevelopmental impairment or mortality^33^. Martin et al. reported that major fluctuations and reductions in splanchnic oxygenation were more evident in infants with transfusion-related NEC^34^.

In the present study, no infant developed transfusion-related NEC, possibly because the cohort had a higher gestational age despite lower pre-transfusion hemoglobin values. Long-term follow-up was not available.

Before transfusion, low hemoglobin levels were associated with a relationship between splanchnic oxygenation and SVC measures reflecting cardiac output. After transfusion, with higher hemoglobin levels, cerebral oxygenation became more closely related to SVC metrics. This suggests that CFTOE and SFTOE vary with changes in hemoglobin level and hemodynamics.

We also assessed correlations between delta changes in hemodynamic variables and oxygenation measures. Delta change in SVCF was significantly correlated with splanchnic oxygenation metrics, and delta change in heart rate was significantly correlated with both cerebral and splanchnic oxygenation measures.

Some infants tolerate low hemoglobin levels without clinical manifestations, whereas others develop symptoms at the same hemoglobin level. Responses to anemia also vary: some infants show increased SVCF, others increased heart rate, some elevated cerebral or splanchnic flow, and others increased FTOE.

These findings suggest that integrating hemodynamic and oxygenation parameters could provide a more comprehensive way to identify infants who are most likely to benefit from transfusion. Future studies with a control group, a larger sample size, and derivation of cut-off values based on integrated parameters are needed.

### Strengths

A major strength of this study is its integrated assessment of hematological, hemodynamic, and oxygenation responses, together with clinical outcomes. This comprehensive approach provides a broader perspective on how transfusion influences multiple physiological domains in preterm infants and may support more individualized decision-making in neonatal care.

### Limitations

Long-term follow-up for neurodevelopmental outcomes was not available.

The relatively small sample size and lack of a control group make it difficult to distinguish transfusion effects from other physiological changes over time.

Neonatal echocardiography and Doppler imaging were performed by a single operator who was not blinded to transfusion status, although the recorded images were reviewed by a consultant radiologist and a consultant neonatologist who were unaware of transfusion status.

Celiac artery flow rather than superior mesenteric artery flow was measured. Although the superior mesenteric artery is a key supplier to the midgut, the celiac artery was selected because it was more accessible for consistent, high-quality insonation in very small preterm infants with abdominal distension or gas, and because celiac artery flow has been used as an indicator of overall splanchnic perfusion changes.

## Conclusion

- SpO2, cerebral and splanchnic oxygenation metrics, SVC diameter, SVC VTI, SVCF, ACA PSV, ACA EDV, and celiac artery PSV showed significant alterations following PRBC transfusion in anemic preterm infants.
- SVCF was strongly correlated with splanchnic oxygenation metrics before transfusion, and SVC VTI was strongly correlated with cerebral oxygenation metrics after transfusion.
- Delta changes in SVC measures were correlated with splanchnic oxygenation metrics, and heart rate was an important clinical monitoring variable in relation to cerebral and splanchnic oxygenation responses.
- The findings support further investigation of an integrated scoring system based on cerebral and splanchnic flow, systemic flow measures such as SVCF and/or cardiac output, and oxygenation parameters to identify infants most likely to benefit from transfusion.
- Symptomatic and asymptomatic anemic infants did not show clear differences in hemodynamic and regional oxygenation responses before and after PRBC transfusion.
- Oxygenation improved significantly after approximately 8 mL/kg of transfused PRBCs had been administered, suggesting that 15 mL/kg, or possibly even less, may be sufficient to improve both cerebral and splanchnic oxygenation.

## Supplementary Information

Below is the link to the electronic supplementary material.

Supplementary Material 1

Supplementary Material 2

Supplementary Material 3

Supplementary Material 4

Supplementary Material 5

Supplementary Material 6

## Data Availability

The datasets generated and/or analyzed during the current study are available from the corresponding author on reasonable request.

## References

[1] Deschmann, E. et al. Clinical practice guideline for red blood cell transfusion thresholds in very preterm neonates. *JAMA Netw. Open* **7**(6), 2417431 (2024). 10.1001/jamanetworkopen.2024.17431 38874929

[2] Bellach, L. et al. Packed red blood cell transfusion in preterm infants. *Lancet Haematol.* **9**(8), 615-e626 (2022). 10.1016/S2352-3026(22)00207-1 35901846

[3] Fredrickson, L. K. et al. Acute physiological effects of packed red blood cell transfusion in preterm infants with different degrees of anaemia. *Arch. Dis. Child. Fetal Neonatal Ed.* **96**(4), F249–F253 (2011). 21097838 10.1136/adc.2010.191023 PMC3114194

[4] Franz, A. R. et al. Effects of liberal vs restrictive transfusion thresholds on survival and neurocognitive outcomes in extremely low-birth-weight infants: the ETTNO randomized clinical trial. *JAMA* **324**(6), 560–570 (2020). 32780138 10.1001/jama.2020.10690 PMC7420159

[5] Hickey, M., Samuels, N., Randive, N., Langford, R. & Kyriacou, P. A. A new fibre optic pulse oximeter probe for monitoring splanchnic organ arterial blood oxygen saturation. *Comput. Methods Programs Biomed.* **108**(3), 883–888 (2012). 21550683 10.1016/j.cmpb.2011.03.019

[6] Zhong, W., Ji, Z. & Sun, C. A review of monitoring methods for cerebral blood oxygen saturation. *Healthcare* **9**(9), 1104 (2021). 34574878 10.3390/healthcare9091104 PMC8466732

[7] Sandal, G. et al. Blood transfusion and tissue oxygenation. *Transfusion* **54**, 1100–1105 (2014). 23901886 10.1111/trf.12359

[8] Kim, D. H. Transfusion practice in neonates. *Korean J. Pediatr.* **61**(9), 265–270 (2018). 30185018 10.3345/kjp.2018.06849 PMC6172519

[9] Jobe, A. H. & Bancalari, E. Bronchopulmonary dysplasia. *Am. J. Respir. Crit. Care Med.* **163**(7), 1723–1729 (2001). 11401896 10.1164/ajrccm.163.7.2011060

[10] Bell, M. J. et al. Neonatal necrotizing enterocolitis. Therapeutic decisions based upon clinical staging. *Ann. Surg.* **187**(1), 1–7 (1978). 413500 10.1097/00000658-197801000-00001 PMC1396409

[11] International Committee for the Classification of Retinopathy of Prematurity. The international classification of retinopathy of prematurity revisited. *Arch. Ophthalmol.* **123**(7), 991–999 (2005). 16009843 10.1001/archopht.123.7.991

[12] Kluckow, M. & Evans, N. Superior vena cava flow in newborn infants: A novel marker of systemic blood flow. *Arch. Dis. Child. Fetal Neonatal Ed.* **82**(3), F182–F187 (2000). 10794783 10.1136/fn.82.3.F182 PMC1721083

[13] Suppan, E., Pichler, G., Binder-Heschl, C., Schwaberger, B. & Urlesberger, B. Three physiological components that influence regional cerebral tissue oxygen saturation. *Front. Pediatr.* **10**, 913223 (2022). 35769216 10.3389/fped.2022.913223 PMC9234387

[14] Basnet, A. & Rout, P. Calculating Fick cardiac output and input. In *StatPearls* (ed. Basnet, A.) (StatPearls Publishing, 2025). 39163462

[15] Yamamoto, A., Burioka, N., Eto, A., Amisaki, T. & Shimizu, E. Usefulness of pulse oximeter that can measure SpO2 to one digit after decimal point. *Yonago Acta Med.* **60**(2), 133–134 (2017). 28701897 PMC5502226

[16] Banerjee, J., Leung, T. S. & Aladangady, N. Cerebral blood flow and oximetry response to blood transfusion in relation to chronological age in preterm infants. *Early Hum. Dev.* **97**, 1–8 (2016). 26619762 10.1016/j.earlhumdev.2015.10.017

[17] Farag, M. M. et al. Hemodynamically significant anemia as an indication of transfusion in preterm infants. *Ital. J. Pediatr.* **51**(1), 140 (2025). 40380201 10.1186/s13052-025-01978-w PMC12084977

[18] Chetan, C. et al. Cerebral hemodynamics in stable preterm infants before and after packed cell transfusion. *J. Nepal Paediatr. Soc.* **41**(3), 353–357 (2021).

[19] Dani, C. et al. Effect of blood transfusions on cerebral haemodynamics in preterm infants. *Acta Paediatr.* **91**(9), 938–941 (2002). 12412869 10.1080/080352502760272623

[20] Krimmel, G. A., Baker, R. & Yanowitz, T. D. Blood transfusion alters the superior mesenteric artery blood flow velocity response to feeding in premature infants. *Am. J. Perinatol.* **26**(2), 99–105 (2009). 19021097 10.1055/s-0028-1090595

[21] Mukhtar, A. Modulation of splanchnic circulation: Role in perioperative management of liver transplant patients. *World J. Gastroenterol.* **22**(4), 1582–1590 (2016). 26819524 10.3748/wjg.v22.i4.1582 PMC4721990

[22] Osada, T. Determination for the comprehensive arterial inflows in the lower abdomen assessed by Doppler ultrasound: methodology, physiological validity and perspective. In *Medical Imaging in Clinical Practice* (ed. Osada, T.) (IntechOpen, 2013).

[23] Farag, M. M., Thabet, M. A. E. H., Abd-Almohsen, A. M. & Ibrahim, H. I. A. M. The effect of placental transfusion on hemodynamics in premature newborns: A randomized controlled trial. *Eur. J. Pediatr.* **181**(12), 4121–4133 (2022). 36129535 10.1007/s00431-022-04619-0 PMC9649456

[24] Farag, M. M., Gouda, M. H., Almohsen, A. M. A. & Khalifa, M. A. Intraventricular hemorrhage prediction in premature neonates in the era of hemodynamics monitoring: A prospective cohort study. *Eur. J. Pediatr.* **181**(12), 4067–4077 (2022). 36171508 10.1007/s00431-022-04630-5 PMC9649466

[25] Farag, M. M., Hassan, M. A. A., Fasseeh, N. A. E. M. & Ghazal, H. A. E. R. The effect of NHFOV on hemodynamics in mild and moderately preterm neonates: A randomized clinical trial. *Eur. J. Pediatr.* **183**(8), 3263–3275 (2024). 38703279 10.1007/s00431-024-05515-5 PMC11263252

[26] de Waal, K. & Kluckow, M. Superior vena cava flow: Role, assessment and controversies in the management of perinatal perfusion. *Semin. Fetal. Neonatal. Med.* **25**(5), 101122 (2020). 32467039 10.1016/j.siny.2020.101122

[27] Farag, M. M., Ghazal, H. A., Ibrahim, A. & Hammad, B. S. Near-infrared spectroscopy measured cerebral oxygenation in full-term infants during transition: An observational study. *Egypt. Pediatr. Assoc. Gaz.* **70**, 53 (2022).

[28] Farag, M. M., Khedr, A. A. E. A. E., Attia, M. H. & Ghazal, H. A. E. Role of near-infrared spectroscopy in monitoring the clinical course of asphyxiated neonates treated with hypothermia. *Am. J. Perinatol.* **41**(4), 429–438 (2024). 34965589 10.1055/s-0041-1740513

[29] Chen, R. et al. Cerebral and intestinal oxygen saturation of different volumes of red blood cell transfusion in preterm infants. *Transfus. Apher. Sci.* **62**(6), 103839 (2023). 37891133 10.1016/j.transci.2023.103839

[30] Van Hoften, J. C., Verhagen, E. A., Keating, P., ter Horst, H. J. & Bos, A. F. Cerebral tissue oxygen saturation and extraction in preterm infants before and after blood transfusion. *Arch. Dis. Child. Fetal. Neonatal. Ed.* **95**(5), F352–F358 (2010). 20466739 10.1136/adc.2009.163592

[31] Bailey, S. M., Hendricks-Muñoz, K. D., Wells, J. T. & Mally, P. Packed red blood cell transfusion increases regional cerebral and splanchnic tissue oxygen saturation in anemic symptomatic preterm infants. *Am. J. Perinatol.* **27**(6), 445–453 (2010). 20099219 10.1055/s-0030-1247598

[32] White, L., Said, M. & Rais-Bahrami, K. Monitoring mesenteric tissue oxygenation with near-infrared spectroscopy during packed red blood cell transfusion in preterm infants. *J. Neonatal. Perinat. Med.* **8**(2), 157–163 (2015). 10.3233/NPM-15814090 26410441

[33] Chock, V. Y. et al. Tissue oxygenation changes after transfusion and outcomes in preterm infants: a secondary near-infrared spectroscopy study of the Transfusion of Prematures randomized clinical trial (TOP NIRS). *JAMA Netw. Open* **6**(9), e2334889 (2023). 37733345 10.1001/jamanetworkopen.2023.34889 PMC10514737

[34] Martin, T. et al. Red blood cell transfusion-related necrotizing enterocolitis in very-low-birthweight infants: A near-infrared spectroscopy investigation. *Transfusion* **53**(11), 2650–2658 (2013). 23480548 10.1111/trf.12158 PMC3686850

