# Supplementary material for: Integrated cerebro-splanchnic blood flow and regional oxygenation monitoring in transfused anemic preterm infants
Source: Sci Rep. 2026 Jun 23;16:19566. doi: 10.1038/s41598-026-53147-6 (PMC13294342; doi:10.1038/s41598-026-53147-6)
Supplement: Supplementary file 1 — Supplementary Material 1 [file 41598_2026_53147_MOESM1_ESM.docx]

**S- figure1: Flow chart of patients.**

**Infant ≤32 weeks admitted to NICU, from March to December 2023**

N=252

**Infant ≤32 weeks having late anemia (onset >3weeks) requiring RBCs transfusion**

N=120

**Infants eligible for analysis (stable preterm infants having late anemia requiring RBCs transfusion)
(*n*= 30):**

-Symptomatic anemia (n=6)

-Asymptomatic anemia (n=24)

**Exclusion (n=90):**

Developed sepsis n=20.

Device unavailability n=30.

Cardiorespiratory instability n=36

Congenital heart D n=4
