# Supplementary material for: Integrated cerebro-splanchnic blood flow and regional oxygenation monitoring in transfused anemic preterm infants
Source: Sci Rep. 2026 Jun 23;16:19566. doi: 10.1038/s41598-026-53147-6 (PMC13294342; doi:10.1038/s41598-026-53147-6)
Supplement: Supplementary file 2 — Supplementary Material 2 [file 41598_2026_53147_MOESM2_ESM.docx]

**S-Table (1a): Neonatal demographics of studied patients.**

| **Variable** | **Number** | **Percent** |
| --- | --- | --- |
| **Sex**  Female  Male | 17  13 | 56.67%  43.33% |
| **GA (weeks)**  Range  Median  IQR  Q1-Q3 | (29-32)  30  1.75  (29.25-31) | |
| **Postnatal age (days )**  Range  Mean  Median  IQR  Q1-Q3 | (21-66)  33.3  31.5  14  (25-39) | |
| **BWT (kg)**  Range  Mean  SD | (0.85-1.7)  1.162  0.2 | |

GA gestational age

BWT birth weight

**S-Table (1b): Maternal and infant characteristics, pregnancy factors, and neonatal outcomes**

| **Variable** | **Number** | **Percent** |
| --- | --- | --- |
| **Parity**  Multi  Primi | 20  10 | 66.67%  33.33% |
| **Fertilization method**  ICSI  Spontaneous | 3  27 | 10%  90% |
| **Multiplicity**  Single  Twin  Triple | 20  9  1 | 66.67%  30%  3.33% |
| **Antenatal care**  No  Yes | 2  28 | 6.67%  93.33% |
| **PET**  No  Yes | 24  6 | 80%  20% |
| **Accidental hemorrhage**  No  Yes | 29  1 | 96.67%  3.33% |
| **PTLP**  No  Yes | 3  27 | 10%  90% |
| **Vaginitis**  No  Yes | 18  12 | 60%  40% |
| **UTI**  No  Yes | 22  8 | 73.33%  26.67% |
| **DM**  No  Yes | 28  2 | 93.33%  6.67% |
| **PROM**  No  Yes | 25  5 | 83.33%  16.67% |
| **Antenatal steroid**  Complete  Incomplete  No | 13  11  6 | 43.33%  36.67%  20% |
| **Mode of delivery**  CS  NVD | 23  7 | 76.67%  23.33% |
| **Resuscitation**  Initial steps  PPV | 16  14 | 53.33%  46.67% |
| **APGAR 1min**  Range  Median  IQR  Q1-Q3 | (4-7)  6  1.75  (5.25-7) | |
| **APGAR 5 min**  Range  Median  IQR  Q1-Q3 | (7-9)  9  1  (8-9) | |

*(ICSI) intracytoplasmic sperm injection; (PET) preeclampsia; (PTLP) preterm labor pain; (UTI) urinary tract infection; (DM) diabetes mellitus; (PROM) premature rupture of membranes; (CS) caesarian section; (NVD) normal vaginal delivery.*

**S-Table (1c): Clinical Outcomes and Interventions.**

| **Variable** | **Number** | **Percent** |
| --- | --- | --- |
| **Cranial US**  IVH G1  IVH G2  IVH G3  No IVH | 8  10  1  11 | 26.67%  33.33%  3.33%  36.67% |
| **PVL**  No  Yes | 28  2 | 93.33%  6.67% |
| **Need for respiratory support**  NCPAP  PTV | 29  1 | 96.67%  3.33% |
| **Need for surfactant**  No  Yes | 29  1 | 96.67%  3.33% |
| **ROP**  No  Yes | 23  7 | 76.67%  23.33% |
| **NEC**  No  Yes | 28  2 | 93.33%  6.67% |
| **BPD**  No  Yes | 27  3 | 90%  10% |
| **Duration of hospital stay**  Range  Mean  SD | (25-69)  48.67  13.3 | |
| **Fate**  Discharge | 30 | 100% |
| **Causes of transfusion**  Symptomatic  Asymptomatic | 24  6 | 80%  20% |
| **No. of blood transfusion**  Range  Median  IQR  Q1-Q3 | (1-6)  3  2  (2-4) | |
| **Caffeine at time of scan** **N=11**  Completing 34week  Reload to treat apnea |  |  |
|  | 8 | 72.7% |
|  | 3 | 27.3% |
| **Blood culture**  Sterile | 30 | 100% |

*(US) Ultrasound; (IVH) intraventricular hemorrhage; (PVL) Periventricular leukomalacia;
(ROP)* *Retinopathy of prematurity; (NEC)* *necrotizing enterocolitis; (BPD)* *bronchopulmonary dysplasia.*

**S-Table (1d): Hematological parameters of studied neonates.**

| **Variable** | **Number** | **Percent** |
| --- | --- | --- |
| **Hb (g/dL)**  Range  Mean  SD | (10.8-18.3)  15.1  1.9 | |
| **HCT (%)**  Range  Mean  SD | (30-53.8)  45.29  5.87 | |
| **MCV (fL)**  Range  Mean  SD | (95-127)  106.9  8.02 | |
| **MCH (pg)**  Range  Mean  SD | (32-43)  37.25  2.7 | |
| **WBCs (n x10^3^ cells/µL)**  Range  Mean  SD | (4.1-28)  12.8  6.4 | |
| **PLT (n x10^3^ cells/µL)**  Range  Mean  SD | (141-490)  267.2  76.65 | |

*(Hb) Hemoglobin; (HCT) Hematocrit;(MCV) Mean corpuscular volume; (MCH)* *Mean corpuscular hemoglobin; (WBCs)* *White blood cell counts; (PLT)* *platelet.*
