# Supplementary material for: Integrated cerebro-splanchnic blood flow and regional oxygenation monitoring in transfused anemic preterm infants
Source: Sci Rep. 2026 Jun 23;16:19566. doi: 10.1038/s41598-026-53147-6 (PMC13294342; doi:10.1038/s41598-026-53147-6)
Supplement: Supplementary file 3 — Supplementary Material 3 [file 41598_2026_53147_MOESM3_ESM.docx]

**S-Table (2a): Comparison of hematological and clinical hemodynamic parameters between asymptomatic and symptomatic infants before transfusion.**

| **Variable** | **Asymptomatic** | **Symptomatic** | | **Sig.** | **P value** |
| --- | --- | --- | --- | --- | --- |
| **Hb (g/dL)**  Range  Median  IQR (Q1-Q3) | (6.5 -7.3)  7.1  0.35 (6.925- 7.275) | (7.1-9.4)  7.85  0.425 (7.6- 8.025) | | 6 | <0.001^*^ |
| **HCT (%)**  Range  Median  IQR (Q1-Q3) | (18.9 -21.3)  19.8  0.72 (19.25- 19.97) | (20.3 -26.5)  22.95  1.4 (22.15- 23.55) | | 1.5 | <0.05* |
| **MCV (fL**)  Range  Median  IQR (Q1-Q3) | (84 -88)  86.5  1.75 (85.25-87) | (82 -93.3)  88  6 (83-89) | | 53 | ˃0.05 |
| **MCH (pg)**  Range  Median  IQR (Q1-Q3) | (32 -34.3)  33.35  1.35 (32.525-33.875) | (30 – 34)  31.45  1.475 (31-32.475) | | 25.5 | 0.01* |
| **WBCs (n x10^3^ cells/µL)**  Range  Median  IQR (Q1-Q3) | (9.7- 15.7)  12.5  2.525 (10.90-13.425) | (4.5- 22.7)  9.4  5.175 (8.05-13.225) | | 44 | ˃0.05 |
| **PLT (n x10^3^ cells/µL)**  Range  Median  IQR (Q1-Q3) | (172- 573)  347.5  236.75 (258- 494.75) | (215 -704)  419  170 (340.5-510.5) | | 58 | ˃0.05 |
| **HR (beat/min)**  Range  Median  IQR (Q1-Q3) | (150 -170)  160  7.5 (152.5-160) | | (130 -180)  170  11.25 (160-171.25) | 37 | ˃0.05 |
| **Systolic Bp (mmHg)**  Range  Median  IQR (Q1-Q3) | (63 -77)  71.5  6.25 (68-74.25) | | (59 – 78)  68  7.75 (63.5-71.25) | 47 | ˃0.05 |
| **Diastolic Bp (mmHg)**  Range  Median  IQR (Q1-Q3) | (32-43)  41  2.75 (39.25-42) | | (30-55)  40  6 (39-45) | 60.5 | ˃0.05 |

**p value (≤0.05) was considered significant. (Hb) Hemoglobin; (HCT) Hematocrit; (MCV) Mean corpuscular volume; (MCH)* *Mean corpuscular hemoglobin; (WBCs)* *White blood cell counts; (PLT)* *platelet. (HR) heart rate; (Bp)blood pressure.*

**S-Table (2b): Comparison of hematological and clinical hemodynamic parameters between asymptomatic and symptomatic infants after transfusion.**

| **Variable** | **Asymptomatic** | **Symptomatic** | **Sig.** | **P value** |
| --- | --- | --- | --- | --- |
| **Hb (g/dL)**  Range  Median  IQR  Q1-Q3 | (9.3 -11.3)  10.4  0.85  (9.9-10.75) | (8.8-11.8)  10  0.9  (9.575-10.475) | 55 | ˃0.05 |
| **HCT (%)**  Range  Median  IQR  Q1-Q3 | (27 -30.8)  29.9  2.3  (27.7-30) | (26- 33.5)  28.25  3.7  (27.15-30.85) | 64 | ˃0.05 |
| **MCV (fL**)  Range  Median  IQR  Q1-Q3 | (84 – 96)  91  6.75  (87.25-94) | (81 – 98)  89  6.75  (85.75-92.5) | 60 | ˃0.05 |
| **MCH (pg)**  Range  Median  IQR  Q1-Q3 | (31 -35.7)  33.6  1.7  (33.05-34.75) | (30 -36.3)  33.05  1.85  (32.15-34) | 60.5 | ˃0.05 |
| **WBCs (n x10^3^ cells/µL)**  Range  Median  IQR  Q1-Q3 | (9.3 -14.2)  12.35  1.9  (11.625- 13.525) | (7.4- 23.5)  11.75  3.575  (9.175- 12.75) | 55.5 | ˃0.05 |
| **PLT (n x10^3^ cells/µL)**  Range  Median  IQR  Q1-Q3 | (219- 659)  330.5  169  (282.75- 451.75) | (216- 573)  379.5  197.25  (294.25- 491.5) | 62 | ˃0.05 |
| **HR (beat/min)**  Range  Median  IQR  Q1-Q3 | (120- 160)  150  0  (150-150) | (120 -160)  140  20  (130-150) | 51 | ˃0.05 |
| **Systolic Bp (mmHg)**  Range  Median  IQR  Q1-Q3 | (57 – 75)  67.5  6.75  (62.25- 69) | (58 – 79)  70  7.25  (65.75- 73) | 47 | ˃0.05 |
| **Diastolic Bp (mmHg)**  Range  Median  IQR  Q1-Q3 | (32 – 43)  40  2.25  (38.5- 40.75) | (35 – 53)  41  4.25  (38- 42.25) | 52.5 | ˃0.05 |
| **Mean Bp** | (42 – 52) | (39 – 60) |  |  |
| Range | 48.5 | 48 | 63 | 0.639 |
| Median | 5.25 | 6.5 |  |  |
| IQR | (44.75- 50) | (45- 51.5) |  |  |

**p value (<0.05) was considered significant using* *Mann Whiteny test ; (Hb)Hemoglobin; (HCT) Hematocrit; (MCV) Mean corpuscular volume; (MCH)* *Mean corpuscular hemoglobin; (WBCs)* *White blood cell counts; (PLT)* *platelet.*

**p value (<0.05) was considered significant using* *Mann Whiteny test; (HR) heart rate; (Bp)blood pressure.*

**S-Table (2c): Comparison of oxygenation parameters in asymptomatic and symptomatic infants prior to blood transfusion.**

| **Variable** | **Asymptomatic** | **Symptomatic** | **Statistical  test value** | **P value** |
| --- | --- | --- | --- | --- |
| **SpO_2_ (%)**  Range  Median  IQR  Q1-Q3 | (95 – 98)  98  0.75  (97.25- 98) | (95 – 98)  96.5  2  (96-98) | 45 | ˃0.05 |
| **Cerebral rSO_2_ (%)** |  |  |  |  |
| Range | (53- 64) | (45 – 73) |  |  |
| Median | 61 | 58.5 | 72 | ˃0.05 |
| IQR | 8 | 6.75 |  |  |
| Q1-Q3 | (55.5-63.5) | (56 – 62.75) |  |  |
| **Cerebral oxygen  consumption (%)**  Range  Median  IQR  Q1-Q3 | (31 – 45)  37  8.75  (33.75- 42.5) | (23 – 53)  37.5  8  (33- 41) | 68.5 | ˃0.05 |
| **Cerebral oxygen  extraction (%)**  Range  Median  IQR  Q1-Q3 | ) 32.6- 45.9(  37.7  8.6  ) 34.675-43.275( | ) 29 – 78(  39.6  7.625  ) 34.7- 42.325( | 66 | ˃0.05 |
| **Intestinal rSO_2_ (%)**  Range  Median  IQR  Q1-Q3 | (15 - 60)  46  14  (42.75-56.75) | (21 – 75)  43  11.25  (38-49.25) | 56 | ˃0.05 |
| **Intestinal oxygen  consumption (%)**  Range  Median  IQR  Q1-Q3 | (35 – 83)  52  14.75  (40.5-55.25) | (21 – 75)  54.5  11.25  (47.75-59) | 58 | ˃0.05 |

**p value (<0.05) was considered significant using* *Mann Whitney test.*

**S-Table (2c): Cont.**

| **Variable** | **Asymptomatic** | **Symptomatic** | **Statistical  test value** | **P value** |
| --- | --- | --- | --- | --- |
| **Intestinal oxygen extraction (%)**  Range  Median  IQR  Q1-Q3 | (36.8- 84.6)  53  14.825  (41.5-56.325) | (21.8 – 78)  55.85  11.1  (48.9-60) | 57.5 | ˃0.05 |
| **SVC diameter (cm)**  Range  Median  IQR  Q1-Q3 | (0.41- 0.58)  0.45  0.0075  (0.45-0.4575) | (0.4- 0.6)  0.46  0.0525  (0.45- 0.5025) | 59 | ˃0.05 |
| **SVC VTI (cm/beat)**  Range  Median  IQR  Q1-Q3 | (9.5 – 13)  11.5  2.1  (10.45- 12.55) | (8.3- 13.6)  11.4  2.1  (10.375- 12.475) | 71.5 | ˃0.05 |
| **SVC outflow (ml/kg/min)**  Range  Median  IQR  Q1-Q3 | (152.5- 596.4)  248.5  76.875  (194.275- 271.150) | (161.7- 524.2)  239.8  101.175  (202.250- 303.425) | 67 | ˃0.05 |
| **ACA(PVs) (cm/sec)**  Range  Median  IQR  Q1-Q3 | (27 – 55)  47.15  16.425  (36.825-54.25) | (32 – 65)  52.5  11  (44-55) | 53.5 | ˃0.05 |
| **(ACA) EDV (cm/sec)**  Range  Median  IQR  Q1-Q3 | (3.2- 10.5)  4.75  2.2  (4.025- 6.225) | (2 -13.7)  6.9  4.25  (5.675- 9.925) | 40.5 | ˃0.05 |
| **(ACA) RI**  Range  Median  IQR  Q1-Q3 | (0.82- 0.92)  0.85  0.03  (0.8425-0.8725) | (0.75 -1.08)  0.84  0.075  (0.8075-0.8825) | 56.5 | ˃0.05 |
| **Celiac A (PVs) (cm/sec)**  Range  Median  IQR  Q1-Q3 | (42.6 -109)  67  24.1  (54.575- 78.675) | (40.3 -136)  69.5  18.75  (58.5-77.25) | 67.5 | ˃0.05 |
| **(Celiac A) EDV (cm/sec)**  Range  Median  IQR  Q1-Q3 | (7 -15.3)  9.8  4.45  (7.8-12.25) | (4 -19.3)  10  4.15  (7.475-11.625) | 66.5 | ˃0.05 |
| **(Celiac A) RI**  Range  Median  IQR  Q1-Q3 | (0.76 -0.9)  0.87  0.035  (0.8525-0.8875) | (0.74- 0.94)  0.84  0.0725  (0.8175-0.89) | 59 | ˃0.05 |

**p value (<0.05) was considered significant using* *Mann Whitney test. (rSO_2_ %) regional tissue oxygenation; (SVC) superior vena cava; (VTI )velocity time integral; (ACA) anterior cerebral artery; (PVs) peak systolic velocity; (EDV) end diastolic velocity; (RI)resistive index; (celiac A) celiac artery.*

**S-Table (3d): Comparative analysis of oxygenation and hemodynamic parameters in asymptomatic versus symptomatic infants after blood transfusion.**

| **Variable** | **Asymptomatic** | **Symptomatic** | **Statistical test value** | **P value** |
| --- | --- | --- | --- | --- |
| **SpO_2_ (%)**  Range  Median  IQR  Q1-Q3 | (97- 98)  98  0.25  (97.25-98) | (97 - 98)  98  0  (98-98) | 60 | ˃0.05 |
| **Cerebral rSO_2_ (%)**  Range  Median  IQR  Q1-Q3 | (68 -78)  75  4.5  (71.25-75.75) | (51- 85)  73  8.75  (68.5-77.25) | 66 | ˃0.05 |
| **Cerebral oxygen  consumption (%)**  Range  Median  IQR  Q1-Q3 | (20-30)  22.5  4  (22-26) | (13 - 46)  25  9  (20.5-29.5) | 65 | ˃0.05 |
| **Cerebral oxygen  extraction (%)**  Range  Median  IQR  Q1-Q3 | (20.4- 30.6)  23  4.25  (22.45-26.7) | (13.2 47.4)  25.45  9.1  (20.925- 30.025) | 65 | ˃0.05 |
| **Intestinal rSO_2_ (%)**  Range  Median  IQR  Q1-Q3 | (41 - 59)  52.5  4  (52-56) | (37 - 75)  54.5  12.25  (50-62.25) | 56.5 | ˃0.05 |
| **Intestinal oxygen  consumption (%)**  Range  Median  IQR  Q1-Q3 | (39 - 56)  45  3.75  (42-45.75) | (22 - 61)  43.5  12.25  (35.75-48) | 57 | ˃0.05 |

**S-Table (3d): Cont.**

| **Variable** | **Asymptomatic** | **Symptomatic** | **Statistical test value** | **P value** |
| --- | --- | --- | --- | --- |
| **Intestinal oxygen extraction (%)**  Range  Median  IQR  Q1-Q3 | (39.7 - 57.7)  46.1  3.925  (42.825-46.75) | (22.6 - 62.2)  44.3  12.45  (36.45-48.9) | 56.5 | ˃0.05 |
| **SVC diameter (cm)**  Range  Median  IQR  Q1-Q3 | (0.4-0.5)  0.45  0.03  (0.420-0.45) | (0.3-0.5)  0.4  0.055  (0.395-0.45) | 72 | ˃0.05 |
| **SVC VTI (cm/beat)**  Range  Median  IQR  Q1-Q3 | (8.2 - 12.4)  10.45  0.85  (10.025-10.875) | (8.4 - 13)  10.9  2.1  (9.35-11.45) | 67.5 | ˃0.05 |
| **SVC outflow (ml/kg/min)**  Range  Median  IQR  Q1-Q3 | (147.7 -268)  184.15  73.5  (154.075-227.575) | (73.7- 227)  135.4  49.2  (116.725-165.925) | 25.5 | 0.01* |
| **ACA(PVs) (cm/sec)**  Range  Median  IQR  Q1-Q3 | (33 - 54)  37.3  5.525  (35.85-41.375) | (26.3 - 51)  38.55  10.575  (34.5-45.075) | 71.5 | ˃0.05 |
| **(ACA)EDV (cm/sec)**  Range  Median  IQR  Q1-Q3 | (4 -9.5)  4.4  3.675  (4.175-7.85) | (2.3 - 11.5)  4.95  2.6  (3.975-6.575) | 66 | ˃0.05 |

**S-table 3d: Cont.**

| **Variable** | **Asymptomatic** | **Symptomatic** | **Statistical test value** | **P value** |
| --- | --- | --- | --- | --- |
| **(ACA)RI**  Range  Median  IQR  Q1-Q3 | (0.79 - 0.88)  0.84  0.0525  (0.8225-0.875) | (0.74 - 0.95)  0.83  0.07  (0.8-0.87) | 72 | ˃0.05 |
| **Celiac A(PVs) (cm/sec)**  Range  Median  IQR  Q1-Q3 | (51 - 86)  61  13.95  (59.5-73.45) | (38- 97.6)  58.95  18.325  (51.25- 69.575) | 51 | ˃0.05 |
| **(Celiac A) EDV (cm/sec)**  Range  Median  IQR  Q1-Q3 | (5.2 - 14.3)  10  3.8  (7.35-11.15) | (6 - 16)  10.3  3.575  (8.225-11.8) | 62.5 | ˃0.05 |
| **(Celiac A) RI**  Range  Median  IQR  Q1-Q3 | (0.8 - 0.89)  0.84  0.0325  (0.815-0.8475) | (0.76 - 0.9)  0.82  0.0625  (0.8075-0.87) | 72 | ˃0.05 |

**p value (<0.05) was considered significant using* *Mann Whitney test; (rSO_2_ %) regional tissue oxygenation; (SVC) superior vena cava; (VTI )velocity time integral; (ACA) anterior cerebral artery; (PVs) peak systolic velocity; (EDV) end diastolic velocity; (RI) resistive index; (celiac A) celiac artery.*
