# Supplementary material for: Integrated cerebro-splanchnic blood flow and regional oxygenation monitoring in transfused anemic preterm infants
Source: Sci Rep. 2026 Jun 23;16:19566. doi: 10.1038/s41598-026-53147-6 (PMC13294342; doi:10.1038/s41598-026-53147-6)
Supplement: Supplementary file 4 — Supplementary Material 4 [file 41598_2026_53147_MOESM4_ESM.docx]

**Table (3a): Correlation between hemodynamics and oxygenation metrics Before PRBCs-transfusion (n = 30).**

|  |  | **HR** | **HB** | **HCT** | **SVC diameter** | **SVC VTI (cm/beat)** | **SVC outflow (ml/kg/min.)** | **ACA** | | | **Celiac A** | | |
| --- | --- | --- | --- | --- | --- | --- | --- | --- | --- | --- | --- | --- | --- |
|  |  |  |  |  |  |  |  | **PSV** | **EDV** | **RI** | **PSV** | **EDV** | **RI** |
| **HR** | **r** |  | 0.025 | 0.154 | 0.068 | 0.031 | 0.231 | 0.203 | 0.409^*^ | -0.276 | -0.270 | -0.068 | -0.261 |
|  | **p** |  | 0.895 | 0.418 | 0.719 | 0.872 | 0.219 | 0.283 | 0.025^*^ | 0.140 | 0.149 | 0.721 | 0.164 |
| **HB** | **r** |  |  | 0.738^*^ | -0.046 | 0.066 | -0.007 | -0.129 | -0.073 | 0.067 | 0.052 | 0.220 | -0.234 |
|  | **p** |  |  | <0.001^*^ | 0.808 | 0.730 | 0.969 | 0.497 | 0.702 | 0.727 | 0.784 | 0.243 | 0.213 |
| **HCT** | **r** |  |  |  | 0.122 | -0.311 | -0.093 | 0.115 | -0.017 | 0.059 | -0.128 | -0.080 | -0.129 |
|  | **p** |  |  |  | 0.522 | 0.094 | 0.626 | 0.544 | 0.931 | 0.755 | 0.502 | 0.675 | 0.497 |
| **SpO2** | **r** | 0.362^*^ | -0.326 | -0.238 | 0.026 | 0.169 | 0.206 | 0.369^*^ | 0.099 | -0.083 | -0.259 | -0.165 | -0.047 |
|  | **p** | 0.049^*^ | 0.079 | 0.206 | 0.893 | 0.373 | 0.274 | 0.045^*^ | 0.601 | 0.664 | 0.167 | 0.384 | 0.803 |
| **Cerebral rSO2** | **r** | -0.213 | 0.023 | -0.026 | 0.165 | 0.356 | 0.151 | -0.254 | 0.145 | -0.039 | -0.304 | -0.010 | -0.285 |
|  | **p** | 0.259 | 0.902 | 0.891 | 0.384 | 0.054 | 0.426 | 0.175 | 0.446 | 0.837 | 0.102 | 0.960 | 0.127 |
| **Cerebral SPO2-rSO2** | **r** | 0.261 | -0.078 | -0.017 | -0.148 | -0.301 | -0.104 | 0.300 | -0.117 | 0.022 | 0.237 | -0.020 | 0.257 |
|  | **p** | 0.164 | 0.680 | 0.928 | 0.434 | 0.106 | 0.584 | 0.107 | 0.538 | 0.908 | 0.207 | 0.918 | 0.171 |
| **Cerebral oxygen extraction** | **r** | 0.281 | 0.521^*^ | 0.436^*^ | -0.043 | -0.120 | 0.094 | -0.065 | -0.183 | -0.019 | 0.000 | 0.216 | -0.230 |
|  | **p** | 0.132 | 0.003^*^ | 0.016^*^ | 0.822 | 0.529 | 0.622 | 0.733 | 0.332 | 0.920 | 0.998 | 0.252 | 0.222 |
| **Intestinal rSO2** | **r** | -0.150 | -0.104 | -0.049 | -0.451^*^ | -0.354 | -0.544^*^ | 0.055 | -0.224 | 0.387^*^ | 0.274 | 0.305 | 0.001 |
|  | **p** | 0.430 | 0.584 | 0.798 | 0.012^*^ | 0.055 | 0.002^*^ | 0.774 | 0.235 | 0.035^*^ | 0.143 | 0.101 | 0.996 |
| **Intestinal SPO2-rSO2** | **r** | 0.182 | 0.068 | 0.023 | 0.441^*^ | 0.361^*^ | 0.549^*^ | -0.016 | 0.227 | -0.384^*^ | -0.293 | -0.313 | -0.006 |
|  | **p** | 0.335 | 0.721 | 0.903 | 0.015^*^ | 0.049^*^ | 0.002^*^ | 0.934 | 0.227 | 0.036^*^ | 0.117 | 0.092 | 0.976 |
| **Intestinal oxygen extraction** | **r** | 0.164 | 0.088 | 0.039 | 0.446^*^ | 0.359 | 0.544^*^ | -0.032 | 0.231 | -0.390^*^ | -0.288 | -0.309 | -0.006 |
|  | **p** | 0.388 | 0.645 | 0.838 | 0.013^*^ | 0.051 | 0.002^*^ | 0.868 | 0.220 | 0.033^*^ | 0.123 | 0.096 | 0.974 |

**r: Pearson coefficient**

*: Statistically significant at p ≤ 0.05

**Table (3b): Correlation between hemodynamics and oxygenation metrics after** **PRBCs-transfusion (n = 30).**

|  |  | **HR** | **HB** | **HCT** | **SVC diameter** | **SVC VTI (cm/beat)** | **SVC outflow (ml/kg/min.)** | **ACA** | | | **Celiac A** | | |
| --- | --- | --- | --- | --- | --- | --- | --- | --- | --- | --- | --- | --- | --- |
|  |  |  |  |  |  |  |  | **PSV** | **EDV** | **RI** | **PSV** | **EDV** | **RI** |
| **HR** | **r** |  | -0.345 | -0.281 | -0.274 | -0.564^*^ | 0.185 | -0.082 | 0.009 | 0.025 | -0.027 | -0.023 | -0.132 |
|  | **p** |  | 0.062 | 0.132 | 0.143 | 0.001^*^ | 0.329 | 0.665 | 0.963 | 0.894 | 0.889 | 0.903 | 0.487 |
| **HB** | **r** |  |  | 0.419^*^ | 0.369^*^ | 0.317 | 0.196 | -0.099 | 0.018 | -0.060 | 0.003 | 0.030 | -0.097 |
|  | **p** |  |  | 0.021^*^ | 0.045^*^ | 0.087 | 0.300 | 0.604 | 0.926 | 0.754 | 0.986 | 0.873 | 0.609 |
| **HCT** | **r** |  |  |  | 0.180 | 0.151 | -0.076 | 0.001 | -0.282 | 0.097 | -0.208 | -0.139 | -0.048 |
|  | **p** |  |  |  | 0.341 | 0.425 | 0.692 | 0.995 | 0.131 | 0.610 | 0.271 | 0.463 | 0.801 |
| **SpO2** | **r** | 0.386^*^ | -0.398^*^ | -0.159 | -0.252 | -0.219 | -0.018 | 0.358 | 0.026 | 0.315 | -0.021 | -0.050 | 0.271 |
|  | **p** | 0.035^*^ | 0.029^*^ | 0.401 | 0.179 | 0.245 | 0.924 | 0.052 | 0.892 | 0.090 | 0.911 | 0.792 | 0.148 |
| **Cerebral rSO2** | **r** | -0.104 | 0.176 | -0.072 | 0.229 | 0.352 | 0.247 | -0.060 | 0.188 | -0.075 | 0.081 | 0.385^*^ | 0.046 |
|  | **p** | 0.586 | 0.352 | 0.707 | 0.223 | 0.057 | 0.188 | 0.752 | 0.321 | 0.694 | 0.672 | 0.036^*^ | 0.810 |
| **Cerebral SPO2-rSO2** | **r** | 0.126 | -0.199 | 0.063 | -0.244 | -0.365^*^ | -0.249 | 0.080 | -0.187 | 0.093 | -0.082 | -0.389^*^ | -0.031 |
|  | **p** | 0.508 | 0.291 | 0.741 | 0.193 | 0.047^*^ | 0.184 | 0.673 | 0.323 | 0.626 | 0.666 | 0.034^*^ | 0.872 |
| **Cerebral oxygen extraction** | **r** | 0.120 | -0.194 | 0.065 | -0.242 | -0.362^*^ | -0.249 | 0.075 | -0.185 | 0.087 | -0.083 | -0.388^*^ | -0.035 |
|  | **p** | 0.527 | 0.305 | 0.732 | 0.198 | 0.049^*^ | 0.185 | 0.694 | 0.327 | 0.646 | 0.662 | 0.034^*^ | 0.856 |
| **Intestinal rSO2** | **r** | -0.035 | -0.053 | -0.017 | 0.101 | -0.233 | -0.114 | 0.111 | 0.018 | -0.047 | -0.218 | -0.291 | 0.241 |
|  | **p** | 0.854 | 0.780 | 0.929 | 0.597 | 0.214 | 0.549 | 0.558 | 0.927 | 0.806 | 0.248 | 0.119 | 0.200 |
| **Intestinal SPO2-rSO2** | **r** | 0.052 | 0.036 | 0.010 | -0.112 | 0.224 | 0.113 | -0.096 | -0.016 | 0.061 | 0.217 | 0.288 | -0.229 |
|  | **p** | 0.785 | 0.852 | 0.958 | 0.557 | 0.234 | 0.551 | 0.615 | 0.931 | 0.750 | 0.250 | 0.122 | 0.224 |
| **Intestinal oxygen extraction** | **r** | 0.044 | 0.043 | 0.013 | -0.106 | 0.228 | 0.114 | -0.102 | -0.016 | 0.054 | 0.219 | 0.291 | -0.233 |
|  | **p** | 0.818 | 0.821 | 0.947 | 0.575 | 0.225 | 0.550 | 0.591 | 0.934 | 0.778 | 0.245 | 0.119 | 0.215 |

**r: Pearson coefficient**

*: Statistically significant at p ≤ 0.05

**Table (3c): Correlation between Delta change of** **hemodynamics and oxygenation metrics (n = 30).**

|  |  | **HR** | **HB** | **HCT** | **SVC diameter** | **SVC VTI (cm/beat)** | **SVC outflow (ml/kg/min.)** | **ACA** | | | **Celiac A** | | |
| --- | --- | --- | --- | --- | --- | --- | --- | --- | --- | --- | --- | --- | --- |
|  |  |  |  |  |  |  |  | **PSV** | **EDV** | **RI** | **PSV** | **EDV** | **RI** |
| **HR** | **r_s_** |  | -0.148 | 0.012 | -0.035 | -0.031 | 0.183 | 0.068 | 0.183 | -0.207 | -0.126 | -0.161 | 0.040 |
|  | **p** |  | 0.436 | 0.949 | 0.856 | 0.873 | 0.333 | 0.722 | 0.333 | 0.273 | 0.507 | 0.396 | 0.832 |
| **HB** | **r_s_** |  |  | 0.392^*^ | 0.081 | -0.003 | 0.176 | 0.190 | -0.246 | 0.293 | -0.320 | 0.158 | -0.369^*^ |
|  | **p** |  |  | 0.032^*^ | 0.670 | 0.988 | 0.354 | 0.315 | 0.190 | 0.116 | 0.085 | 0.405 | 0.045^*^ |
| **HCT** | **r_s_** |  |  |  | 0.148 | 0.127 | 0.261 | 0.063 | -0.018 | -0.102 | -0.314 | -0.021 | -0.362^*^ |
|  | **p** |  |  |  | 0.434 | 0.505 | 0.164 | 0.741 | 0.926 | 0.591 | 0.091 | 0.914 | 0.049^*^ |
| **SpO2** | **r_s_** | 0.195 | 0.145 | 0.073 | 0.020 | 0.413^*^ | 0.279 | 0.366^*^ | -0.026 | 0.119 | -0.114 | -0.113 | -0.076 |
|  | **p** | 0.301 | 0.446 | 0.700 | 0.915 | 0.023^*^ | 0.135 | 0.047^*^ | 0.892 | 0.530 | 0.549 | 0.552 | 0.690 |
| **CrSO2** | **r_s_** | -0.518^*^ | 0.156 | -0.162 | -0.106 | -0.146 | -0.291 | -0.197 | -0.001 | 0.002 | -0.176 | 0.159 | 0.048 |
|  | **p** | 0.003^*^ | 0.410 | 0.393 | 0.577 | 0.442 | 0.118 | 0.298 | 0.995 | 0.990 | 0.353 | 0.401 | 0.803 |
| **SPO2-CrSO2** | **r_s_** | 0.531^*^ | -0.127 | 0.187 | 0.067 | 0.156 | 0.286 | 0.200 | -0.033 | 0.027 | 0.152 | -0.190 | -0.028 |
|  | **p** | 0.003^*^ | 0.503 | 0.323 | 0.725 | 0.412 | 0.126 | 0.290 | 0.862 | 0.888 | 0.423 | 0.314 | 0.883 |
| **CFTOE** | **r_s_** | 0.520^*^ | -0.010 | 0.295 | 0.110 | 0.066 | 0.338 | 0.198 | -0.028 | -0.076 | 0.011 | -0.078 | -0.177 |
|  | **p** | 0.003^*^ | 0.957 | 0.114 | 0.564 | 0.729 | 0.068 | 0.294 | 0.882 | 0.691 | 0.954 | 0.681 | 0.351 |
| **SrSO2** | **r_s_** | -0.500^*^ | -0.108 | -0.140 | -0.256 | -0.295 | -0.535^*^ | -0.295 | -0.193 | 0.215 | 0.191 | -0.031 | 0.211 |
|  | **p** | 0.005^*^ | 0.570 | 0.460 | 0.173 | 0.113 | 0.002^*^ | 0.113 | 0.307 | 0.253 | 0.311 | 0.870 | 0.264 |
| **SPO2-SrSO2** | **r_s_** | 0.488^*^ | 0.126 | 0.147 | 0.234 | 0.341 | 0.542^*^ | 0.307 | 0.163 | -0.177 | -0.190 | 0.035 | -0.252 |
|  | **p** | 0.006^*^ | 0.508 | 0.438 | 0.213 | 0.065 | 0.002^*^ | 0.099 | 0.391 | 0.350 | 0.314 | 0.853 | 0.180 |
| **SFTOE** | **r_s_** | 0.493^*^ | 0.114 | 0.164 | 0.233 | 0.328 | 0.526^*^ | 0.288 | 0.179 | -0.213 | -0.207 | 0.026 | -0.228 |
|  | **p** | 0.006^*^ | 0.548 | 0.387 | 0.215 | 0.077 | 0.003^*^ | 0.123 | 0.344 | 0.259 | 0.273 | 0.892 | 0.225 |

**r_s_: Spearman coefficient** *: Statistically significant at p ≤ 0.05

**S rSO2= Splanchnic rSO2, SFTOE= Splanchnic oxygen extraction , Cr SO2= Cerebral rSO2, CFTOE= cerebral oxygen extraction**
