# Supplementary material for: Integrated cerebro-splanchnic blood flow and regional oxygenation monitoring in transfused anemic preterm infants
Source: Sci Rep. 2026 Jun 23;16:19566. doi: 10.1038/s41598-026-53147-6 (PMC13294342; doi:10.1038/s41598-026-53147-6)
Supplement: Supplementary file 5 — Supplementary Material 5 [file 41598_2026_53147_MOESM5_ESM.docx]

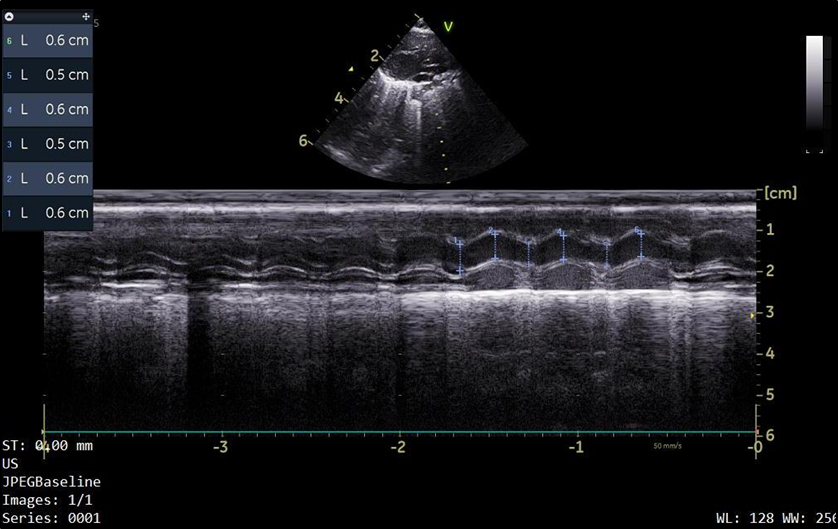


**S-Figure (2):** Superior vena cava diameter in long axis parasternal view by M mode.


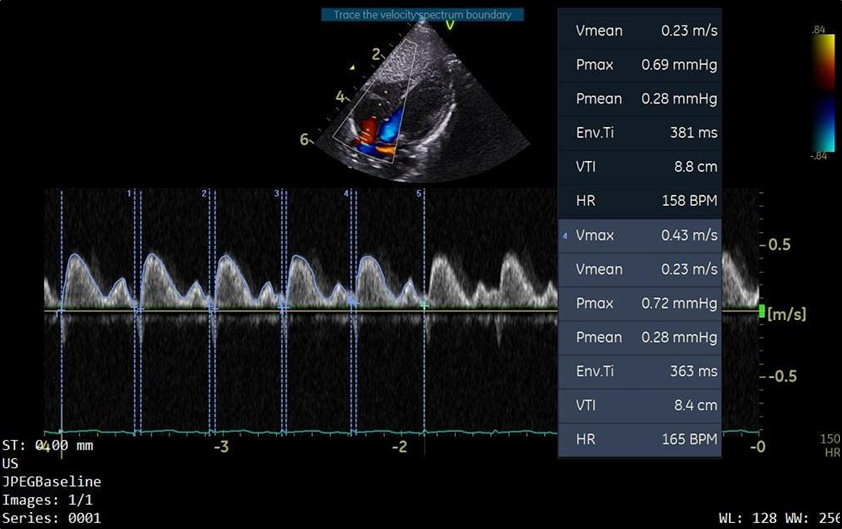


**S-Figure (3):** Pulsed wave Doppler ultrasound study of superior vena cava velocity time integral in low subcostal view.


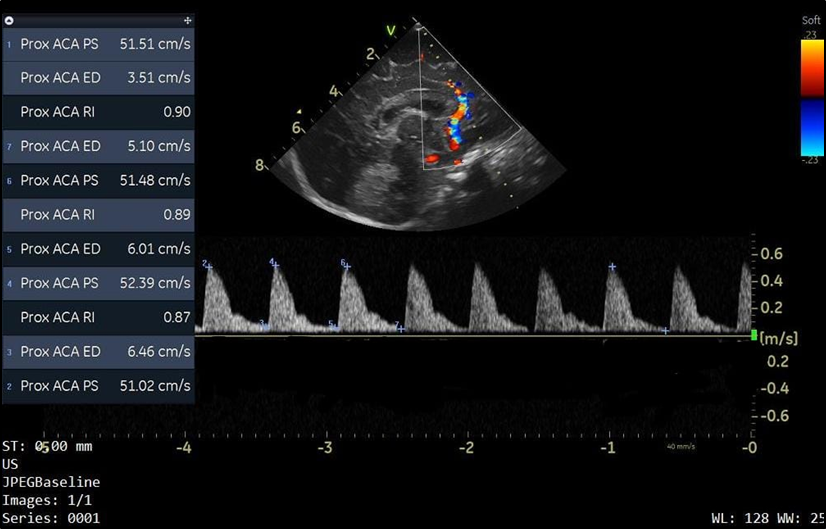


**S-Figure (4):** Pulsed wave Doppler ultrasound study of anterior cerebral artery flow velocity in midsagittal view.


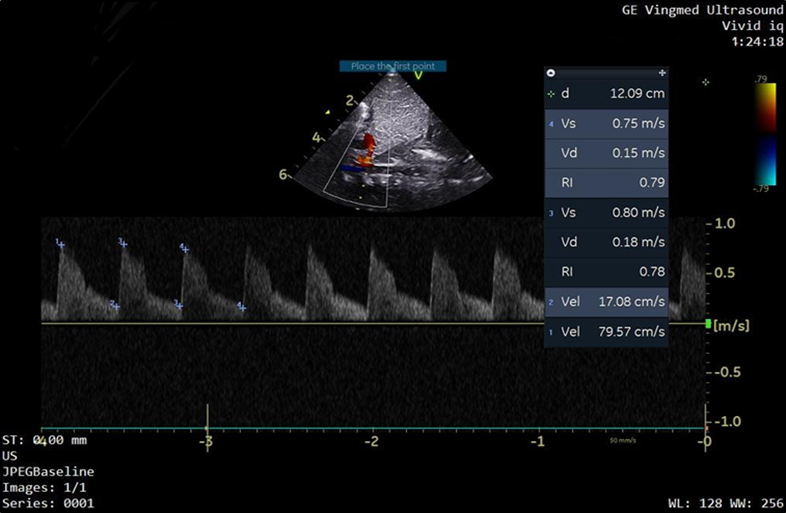


**S-Figure (5):** Pulsed wave doppler ultrasound study of celiac artery flow velocity.


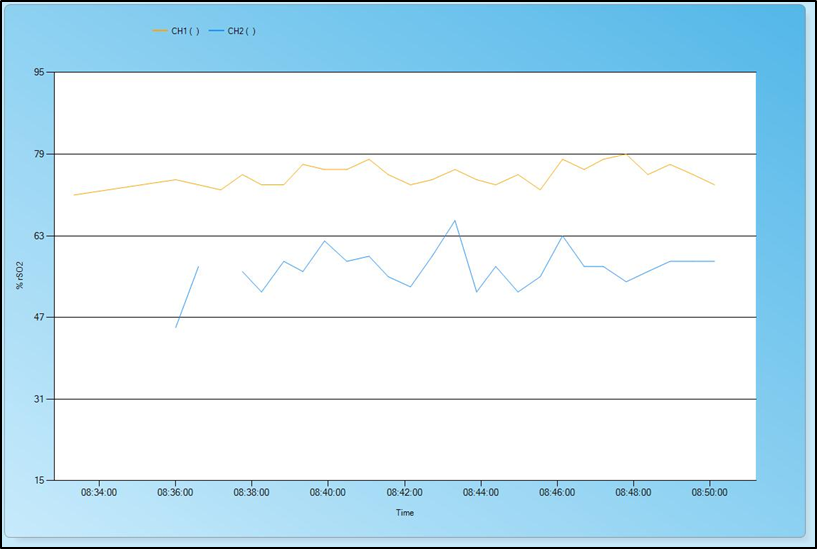


**S-Figure (6):** Regional oxygenation by NIRS, CH_1_ (blue)represent cerebral rSO_2_, CH_2_ (orange) represents intestinal rSO_2_.

**Figures (7 – 11):** show oxygenation and hemodynamic study of 30 weeks preterm infant, born 1 kg with initial hemoglobin 15 gm/dl, hematocrit 44%, needed NCPAP for 3 days, examination was done at day 38 with weight 1.315 kg, hemoglobin 7 gm/dl and hematocrit 19.9% examined by NIRS, echocardiography & doppler ultrasound 30-60 minutes before blood transfusion.


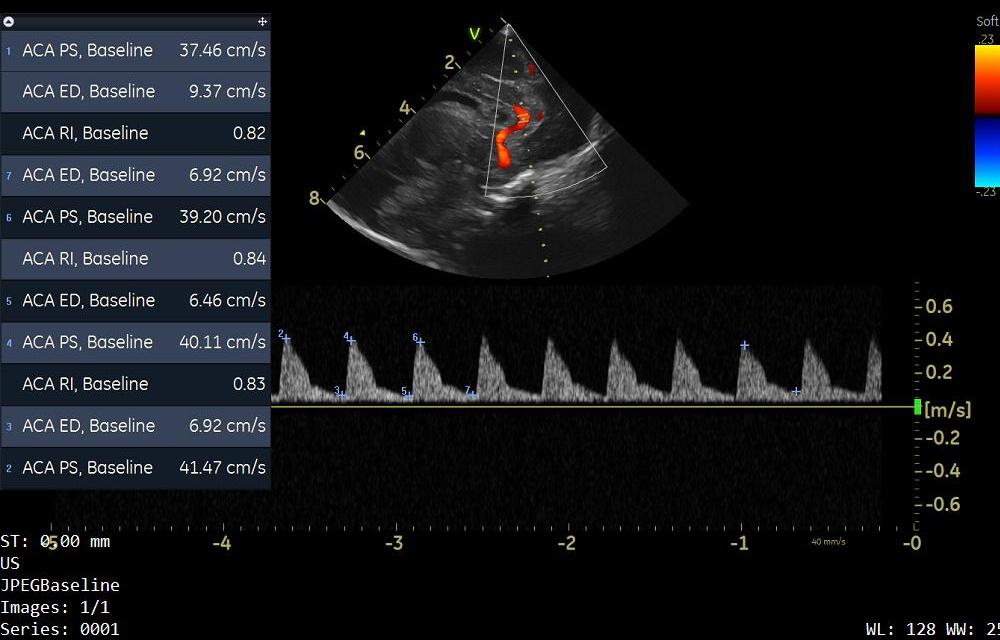


**S-Figure (7):** Pulsed wave Doppler ultrasound was performed to assess blood flow velocity in the ACA in a midsagittal view.

*Three measurements were taken, and the average values were calculated. PSV was recorded as 40.2 cm/sec, maximum EDV as 6.7 cm/sec, and the RI as 0.84. These measurements are significant for evaluating cerebral perfusion in the context of anemia in preterm infants, where compensatory mechanisms may influence blood flow to maintain adequate oxygen delivery.*


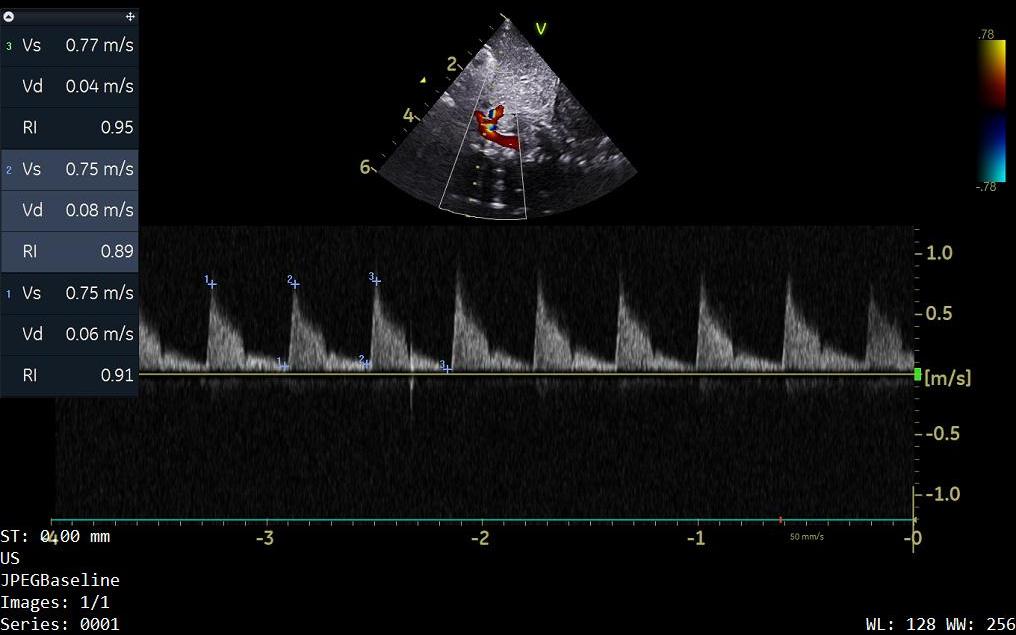


**S-Figure (8):** Celiac artery flow velocity was measured using pulsed wave Doppler ultrasound, with three recordings averaged to calculate mean values.

*The peak systolic velocity was 75.6 cm/sec, maximum end-diastolic velocity was 6 cm/sec, and the RI was 0.91. This data is essential for assessing mesenteric blood flow, which may be compromised in anemic infants due to reduced oxygenation, highlighting the importance of monitoring splanchnic circulation.*


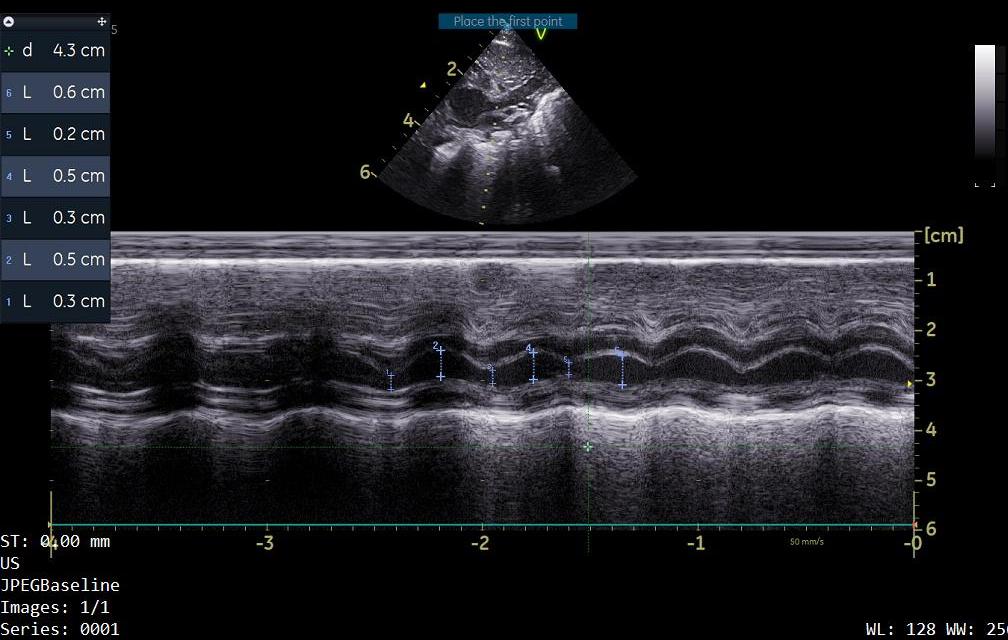


**S-Figure (9):** M-mode ultrasound was used to measure the SVC diameter in the long-axis parasternal view.

*Three minimal and three maximal diameters were recorded during three cardiac cycles, and the mean was calculated. Superior vena cava diameter is a critical indicator of central venous pressure and volume status, which are essential parameters in the management of fluid balance and perfusion in preterm infants, especially those with anemia.*


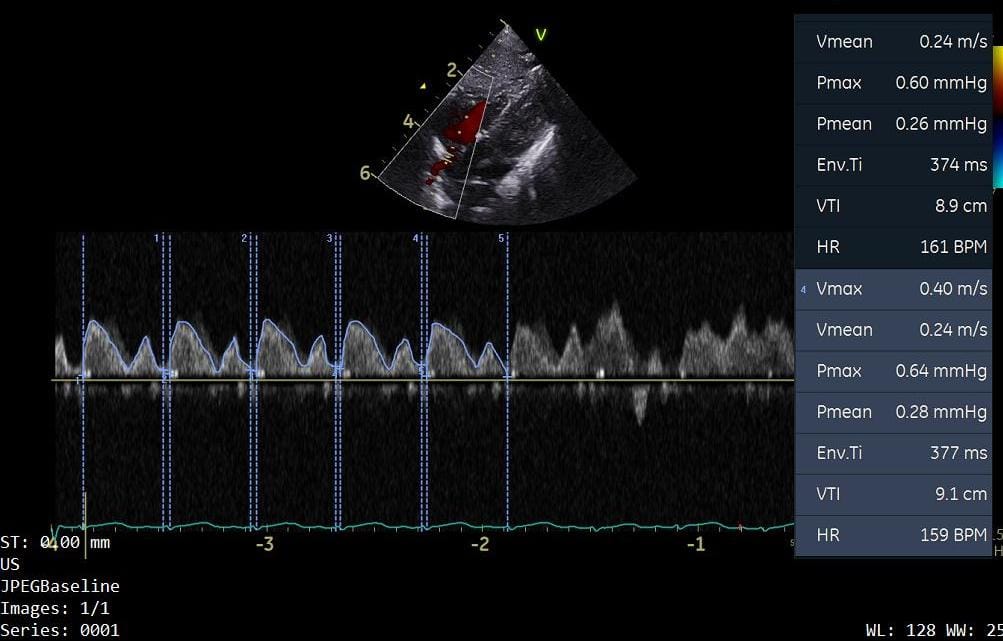


**S-Figure (10):** The VTI of the superior vena cava was measured using pulsed wave Doppler ultrasound in a low subcostal view.

*The mean VTI was calculated from five consecutive cardiac cycles. Using the formula for SVC flow calculation:*

$$SVC flow\text{ (ml/kg/min) }\text{can calculated }= \frac{\left\{ \text{VTI (cm/beat) × 3.14 × (SVC }\text{diameter}^{2}\text{/4) × heart rate (beat/min) } \right\}}{\text{body weight in kg}}$$

$$\frac{\left\{ \text{ }\text{9.5}\text{× 3.14 × ( }\text{0.41}^{2}\text{/4) × }\text{160}\text{ } \right\}}{\text{1.315}}$$

The superior vena cava flow was calculated as 152.5 ml/kg/min. This calculation provides insight into venous return and overall cardiac output, both of which are crucial factors in understanding the hemodynamic state of anemic preterm infants.


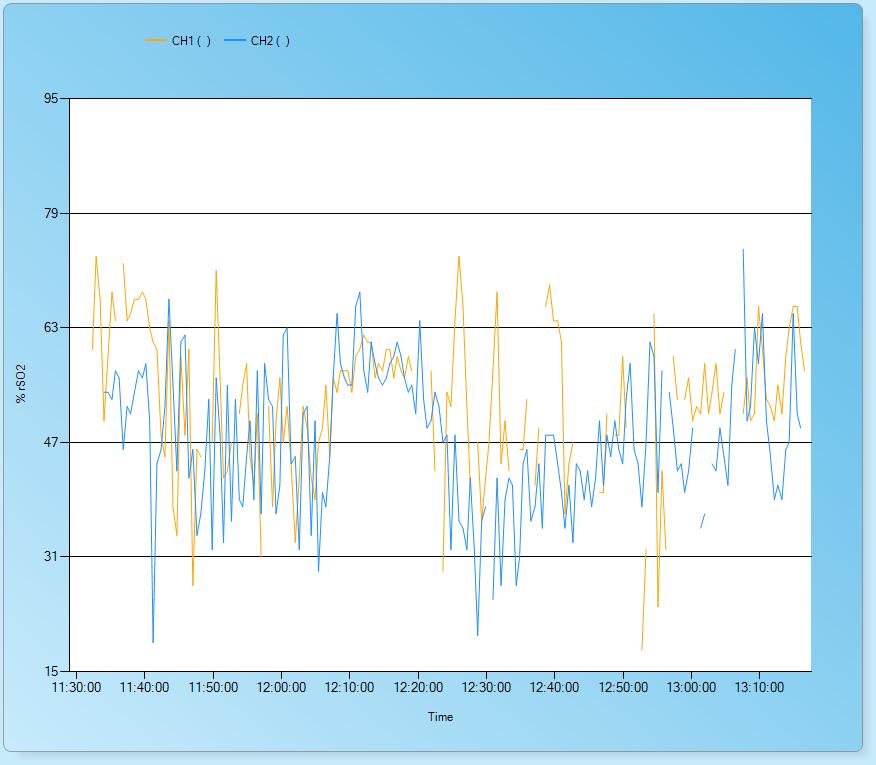


**S-Figure (11):** Regional oxygenation was measured using NIRS.

*Cerebral rSO_2_ was 53%, while intestinal rSO_2_ was 47%. These values reflect tissue oxygenation levels, indicating decreased oxygen delivery to both cerebral and intestinal tissues. In the context of anemia, these findings suggest impaired oxygen supply to vital organs, underscoring the need for blood transfusion to restore adequate tissue oxygenation.*
