# Supplementary material for: Integrated cerebro-splanchnic blood flow and regional oxygenation monitoring in transfused anemic preterm infants
Source: Sci Rep. 2026 Jun 23;16:19566. doi: 10.1038/s41598-026-53147-6 (PMC13294342; doi:10.1038/s41598-026-53147-6)
Supplement: Supplementary file 6 — Supplementary Material 6 [file 41598_2026_53147_MOESM6_ESM.docx]

# s-Table 4a comparison of hemodynamic and oxygenation parameters in patients with and without ROP.

| **Timepoint** | **Variable** | **Mean/median** | **No ROP(n=23)** | **Yes ROP(n=7)** | **Test** | **p value** |
| --- | --- | --- | --- | --- | --- | --- |
| - | Duration of hospital stay (days) | Mean ± SD | 45.83 ± 13.35 | 58.00 ± 8.29 | Welch t-test | **0.010** |
| before | Cerebral_rSO2 | Mean ± SD | 58.74 ± 6.84 | 61.00 ± 3.56 | Welch t-test | 0.262 |
| before | Cerebral-oxygen, consumption | Mean ± SD | 38.04 ± 7.39 | 35.57 ± 3.64 | Welch t-test | 0.245 |
| before | Cerebral-oxygen, extraction | Median (IQR) | 39.70 (36.05–43.80) | 34.70 (34.15–39.35) | Mann–Whitney U | 0.194 |
| before | Intestinal_rSO2 | Mean ± SD | 45.17 ± 10.57 | 40.57 ± 13.43 | Welch t-test | 0.429 |
| before | Intestinal-oxygen, consumption | Mean ± SD | 51.61 ± 10.80 | 56.00 ± 14.15 | Welch t-test | 0.470 |
| before | Intestinal-oxygen, extraction | Mean ± SD | 53.23 ± 11.10 | 57.71 ± 14.12 | Welch t-test | 0.462 |
| before | SVC-diameter | Median (IQR) | 0.45 (0.45–0.50) | 0.50 (0.49–0.55) | Mann–Whitney U | **0.028** |
| before | SVC-VTI cm/beat | Mean ± SD | 11.68 ± 1.32 | 10.60 ± 1.51 | Welch t-test | 0.123 |
| before | SVC-outflow ml/kg/min | Median (IQR) | 232.20 (187.35–305.15) | 271.70 (214.70–273.25) | Mann–Whitney U | 0.413 |
| before | ACA-PSV | Mean ± SD | 49.07 ± 9.66 | 47.23 ± 9.97 | Welch t-test | 0.675 |
| before | ACA-EDV | Mean ± SD | 7.05 ± 2.71 | 7.61 ± 3.85 | Welch t-test | 0.728 |
| before | ACA-RI | Median (IQR) | 0.84 (0.81–0.89) | 0.84 (0.83–0.87) | Mann–Whitney U | 0.922 |
| before | Celiac A-PSV | Median (IQR) | 67.60 (53.50–75.50) | 78.00 (66.00–84.00) | Mann–Whitney U | 0.280 |
| before | Celiac A-EDV | Mean ± SD | 10.33 ± 3.24 | 8.07 ± 2.60 | Welch t-test | 0.082 |
| before | Celiac A-RI | Mean ± SD | 0.84 ± 0.05 | 0.87 ± 0.04 | Welch t-test | 0.114 |
| during | Cerebral-rSO2 | Mean ± SD | 68.96 ± 5.45 | 73.29 ± 5.68 | Welch t-test | 0.106 |
| during | Cerebral-oxygen, consumption | Mean ± SD | 27.57 ± 5.32 | 23.86 ± 5.64 | Welch t-test | 0.155 |
| during | Cerebral-oxygen, extraction | Mean ± SD | 28.50 ± 5.52 | 24.51 ± 5.82 | Welch t-test | 0.140 |
| during | Intestinal-rSO2 | Mean ± SD | 52.43 ± 11.79 | 42.14 ± 7.93 | Welch t-test | **0.018** |
| during | Intestinal-oxygen-consumption | Mean ± SD | 44.13 ± 11.92 | 55.00 ± 8.39 | Welch t-test | **0.017** |
| during | Intestinal-oxygen-extraction | Mean ± SD | 45.60 ± 12.43 | 56.51 ± 8.37 | Welch t-test | **0.018** |
| after | Cerebral_rSO2 | Mean ± SD | 71.48 ± 7.87 | 75.29 ± 4.07 | Welch t-test | 0.106 |
| after | Cerebral-oxygen-consumption | Mean ± SD | 26.26 ± 7.87 | 22.71 ± 4.07 | Welch t-test | 0.130 |
| after | Cerebral-oxygen-extraction | Mean ± SD | 26.82 ± 8.07 | 23.13 ± 4.14 | Welch t-test | 0.124 |
| after | Intestinal_rSO2 | Mean ± SD | 56.22 ± 9.22 | 51.29 ± 8.92 | Welch t-test | 0.232 |
| after | Intestinal-oxygen-consumption | Mean ± SD | 41.52 ± 9.19 | 46.71 ± 8.92 | Welch t-test | 0.210 |
| after | Intestinal-oxygen-extraction | Mean ± SD | 42.42 ± 9.43 | 47.61 ± 9.08 | Welch t-test | 0.218 |
| after | SVC-diameter | Median (IQR) | 0.40 (0.40–0.45) | 0.40 (0.38–0.43) | Mann–Whitney U | 0.629 |
| after | SVC-VTI-cm/beat | Mean ± SD | 10.38 ± 1.35 | 11.07 ± 1.24 | Welch t-test | 0.232 |
| after | SVC outflow ml/kg/min | Median (IQR) | 153.00 (126.70–180.60) | 135.80 (116.15–147.15) | Mann–Whitney U | 0.377 |
| after | ACA-PSV | Mean ± SD | 38.77 ± 6.67 | 40.39 ± 6.60 | Welch t-test | 0.583 |
| after | ACA-EDV | Mean ± SD | 5.51 ± 2.09 | 5.19 ± 3.07 | Welch t-test | 0.801 |
| after | ACA-RI | Mean ± SD | 0.83 ± 0.04 | 0.86 ± 0.07 | Welch t-test | 0.380 |
| after | Celiac A-PSV | Mean ± SD | 60.30 ± 14.33 | 63.86 ± 14.06 | Welch t-test | 0.572 |
| after | Celiac A-EDV | Mean ± SD | 9.97 ± 2.76 | 10.89 ± 3.32 | Welch t-test | 0.524 |
| after | Celiac A_RI | Median (IQR) | 0.82 (0.81–0.86) | 0.87 (0.81–0.88) | Mann–Whitney U | 0.505 |

***Significant p-values are shown in bold red.* *Abbreviation: ROP = retinopathy of prematurity.***

**s-Table 4b comparison of hemodynamic and oxygenation parameters in patients with and without BPD.**

| Timepoint | Variable | Mean/median | BPD No (n=27) | BPD Yes(n=3) | Test | p value |
| --- | --- | --- | --- | --- | --- | --- |
|  | Duration of hospital stay (days) | Mean ± SD | 47.30 ± 13.22 | 61.00 ± 6.08 | Mann-Whitney U | 0.067 |
| before | Cerebral_rSO2 | Mean ± SD | 59.48 ± 6.03 | 57.33 ± 9.29 | Mann-Whitney U | 0.917 |
| before | Cerebral_O2_consumption | Mean ± SD | 37.30 ± 6.66 | 39.00 ± 8.72 | Mann-Whitney U | 0.917 |
| before | Cerebral_O2_extraction | Median (IQR) | 39.50 (34.70-42.45) | 36.80 (35.20-43.90) | Mann-Whitney U | 0.972 |
| before | Intestinal_rSO2 | Mean ± SD | 42.70 ± 10.02 | 56.67 ± 16.07 | Mann-Whitney U | 0.077 |
| before | Intestinal_O2_consumption | Median (IQR) | 55.00 (49.00-59.00) | 45.00 (33.00-49.00) | Mann-Whitney U | 0.077 |
| before | Intestinal_O2_extraction | Mean ± SD | 55.75 ± 10.47 | 41.03 ± 16.99 | Mann-Whitney U | 0.072 |
| before | SVC-diameter | Median (IQR) | 0.45 (0.45-0.51) | 0.42 (0.41-0.46) | Mann-Whitney U | 0.166 |
| before | SVC_VTI | Mean ± SD | 11.52 ± 1.44 | 10.57 ± 0.93 | Mann-Whitney U | 0.268 |
| before | SVC-outflow | Median (IQR) | 258.70 (204.05-305.15) | 172.00 (166.85-191.50) | Mann-Whitney U | 0.041 |
| before | ACA_PSV | Median (IQR) | 52.00 (44.00-55.00) | 37.20 (37.10-45.60) | Mann-Whitney U | 0.316 |
| before | ACA_EDV | Mean ± SD | 7.17 ± 2.64 | 7.27 ± 5.94 | Mann-Whitney U | 0.917 |
| before | ACA_RI | Median (IQR) | 0.84 (0.82-0.88) | 0.83 (0.79-0.89) | Mann-Whitney U | 0.755 |
| before | Celiac-PSV | Mean ± SD | 69.79 ± 15.28 | 74.87 ± 53.09 | Mann-Whitney U | 0.447 |
| before | Celiac-EDV | Mean ± SD | 9.55 ± 2.73 | 12.07 ± 6.62 | Mann-Whitney U | 0.703 |
| before | Celiac-RI | Mean ± SD | 0.85 ± 0.05 | 0.83 ± 0.03 | Mann-Whitney U | 0.367 |
| during | Cerebral_rSO2 | Mean ± SD | 69.67 ± 5.94 | 72.67 ± 1.53 | Mann-Whitney U | 0.225 |
| during | Cerebral_O2_consumption | Mean ± SD | 27.07 ± 5.68 | 23.33 ± 2.08 | Mann-Whitney U | 0.156 |
| during | Cerebral_O2_extraction | Mean ± SD | 27.94 ± 5.92 | 24.23 ± 2.04 | Mann-Whitney U | 0.167 |
| during | Intestinal_rSO2 | Median (IQR) | 48.00 (41.50-55.00) | 80.00 (59.50-80.00) | Mann-Whitney U | 0.268 |
| during | Intestinal_O2_consumption | Median (IQR) | 48.00 (42.00-56.50) | 16.00 (15.50-37.00) | Mann-Whitney U | 0.267 |
| during | Intestinal_O2_extraction | Median (IQR) | 50.00 (43.25-57.50) | 16.00 (15.85-37.85) | Mann-Whitney U | 0.268 |
| after | Cerebral_rSO2 | Mean ± SD | 71.70 ± 7.33 | 78.33 ± 3.51 | Mann-Whitney U | 0.090 |
| after | Cerebral_O2_consumption | Mean ± SD | 26.11 ± 7.29 | 19.33 ± 3.51 | Mann-Whitney U | 0.096 |
| after | Cerebral_O2_extraction | Mean ± SD | 26.65 ± 7.48 | 19.73 ± 3.56 | Mann-Whitney U | 0.090 |
| after | Intestinal_rSO2 | Median (IQR) | 53.00 (50.00-60.00) | 75.00 (66.00-75.00) | Mann-Whitney U | 0.035 |
| after | Intestinal_O2_consumption | Mean ± SD | 44.30 ± 7.82 | 28.67 ± 10.69 | Mann-Whitney U | 0.035 |
| after | Intestinal_O2_extraction | Mean ± SD | 45.23 ± 8.02 | 29.27 ± 10.86 | Mann-Whitney U | 0.035 |
| after | SVC-diameter | Median (IQR) | 0.40 (0.40-0.45) | 0.45 (0.43-0.45) | Mann-Whitney U | 0.390 |
| after | SVC-VTI | Mean ± SD | 10.68 ± 1.32 | 9.30 ± 0.82 | Mann-Whitney U | 0.104 |
| after | SVC-outflow | Mean ± SD | 156.45 ± 45.38 | 129.10 ± 20.83 | Mann-Whitney U | 0.300 |
| after | ACA_PSV | Mean ± SD | 39.16 ± 6.67 | 39.00 ± 7.01 | Mann-Whitney U | 0.945 |
| after | ACA-EDV | Mean ± SD | 5.20 ± 2.06 | 7.50 ± 3.74 | Mann-Whitney U | 0.283 |
| after | ACA-RI | Mean ± SD | 0.84 ± 0.05 | 0.79 ± 0.05 | Mann-Whitney U | 0.110 |
| after | Celiac-PSV | Mean ± SD | 61.15 ± 12.01 | 60.93 ± 31.79 | Mann-Whitney U | 0.533 |
| after | Celiac-EDV | Mean ± SD | 10.28 ± 3.00 | 9.30 ± 1.18 | Mann-Whitney U | 0.628 |
| after | Celiac-RI | Median (IQR) | 0.82 (0.81-0.87) | 0.87 (0.83-0.88) | Mann-Whitney U | 0.626 |

***Significant p-values (<0.05) are shown in bold red. BPD bronchopulmonary dysplasia***

**s-Table 4c comparison of hemodynamic and oxygenation parameters in patients with and without NEC.**

| **Variable** | **NEC No (n=28)** | **NEC Yes (n=2)** | **Test** | **p value** |
| --- | --- | --- | --- | --- |
| Hospital stay (days) | 50.00 (36.50–57.25) | 61.50 (58.25–64.75) | Mann–Whitney U (exact) | 0.1655 |
| Cerebral rSO2 - before | 58.50 (56.00–64.00) | 61.00 (60.50–61.50) | Mann–Whitney U (exact) | 0.6621 |
| Cerebral rSO2 - during | 70.00 (66.50–74.25) | 70.00 (69.50–70.50) | Mann–Whitney U (exact) | 0.9011 |
| Cerebral rSO2 - after | 73.00 (68.75–77.25) | 75.00 (74.50–75.50) | Mann–Whitney U (exact) | 0.7172 |
| Cerebral oxygen consumption - before | 37.50 (33.00–41.00) | 36.00 (35.00–37.00) | Mann–Whitney U (exact) | 0.8368 |
| Cerebral oxygen consumption - during | 27.00 (22.75–30.00) | 26.00 (26.00–26.00) | Mann–Whitney U (exact) | 0.7172 |
| Cerebral oxygen consumption - after | 25.00 (20.75–29.25) | 22.50 (22.25–22.75) | Mann–Whitney U (exact) | 0.6621 |
| Cerebral oxygen extraction - before | 39.60 (34.53–42.73) | 37.05 (36.23–37.88) | Mann–Whitney U (exact) | 0.6069 |
| Cerebral oxygen extraction - during | 28.10 (23.20–31.05) | 27.05 (26.93–27.18) | Mann–Whitney U (exact) | 0.7172 |
| Cerebral oxygen extraction - after | 25.45 (21.15–29.77) | 23.05 (22.72–23.38) | Mann–Whitney U (exact) | 0.6621 |
| Intestinal rSO2 - before | 43.50 (38.75–50.00) | 27.00 (21.00–33.00) | Mann–Whitney U (exact) | 0.1149 |
| Intestinal rSO2 - during | 51.00 (41.75–56.50) | 41.50 (40.75–42.25) | Mann–Whitney U (exact) | 0.2575 |
| Intestinal rSO2 - after | 54.50 (50.75–61.25) | 45.00 (41.00–49.00) | Mann–Whitney U (exact) | 0.2253 |
| Intestinal oxygen consumption - before | 53.50 (47.00–57.50) | 70.00 (63.50–76.50) | Mann–Whitney U (exact) | 0.1149 |
| Intestinal oxygen consumption - during | 46.00 (40.25–56.25) | 54.50 (53.25–55.75) | Mann–Whitney U (exact) | 0.3310 |
| Intestinal oxygen consumption - after | 43.50 (36.75–47.25) | 52.50 (48.25–56.75) | Mann–Whitney U (exact) | 0.2575 |
| Intestinal oxygen extraction - before | 54.80 (48.77–60.00) | 71.95 (65.62–78.27) | Mann–Whitney U (exact) | 0.1149 |
| Intestinal oxygen extraction - during | 47.40 (41.40–57.25) | 56.70 (55.70–57.70) | Mann–Whitney U (exact) | 0.2575 |
| Intestinal oxygen extraction - after | 44.30 (37.45–48.15) | 53.75 (49.52–57.98) | Mann–Whitney U (exact) | 0.2253 |
| SVC diameter - before | 0.45 (0.45–0.50) | 0.54 (0.52–0.56) | Mann–Whitney U (exact) | 0.1931 |
| SVC diameter - after | 0.40 (0.40–0.45) | 0.36 (0.33–0.40) | Mann–Whitney U (exact) | 0.4138 |
| SVC VTI - before | 11.20 (10.28–12.47) | 12.55 (12.43–12.68) | Mann–Whitney U (exact) | 0.3310 |
| SVC VTI - after | 10.55 (9.35–11.45) | 10.90 (10.75–11.05) | Mann–Whitney U (exact) | 0.8368 |
| SVC outflow - before | 229.10 (195.95–272.88) | 449.05 (375.38–522.73) | Mann–Whitney U (exact) | 0.0736 |
| SVC outflow - after | 147.15 (123.18–177.10) | 137.50 (126.25–148.75) | Mann–Whitney U (exact) | 0.6621 |
| ACA PSV - before | 52.50 (43.58–55.00) | 37.00 (32.00–42.00) | Mann–Whitney U (exact) | 0.1655 |
| ACA PSV - after | 38.75 (35.55–45.08) | 35.80 (34.70–36.90) | Mann–Whitney U (exact) | 0.4598 |
| ACA EDV - before | 6.70 (4.95–9.45) | 7.60 (5.40–9.80) | Mann–Whitney U (exact) | 1.0000 |
| ACA EDV - after | 4.40 (4.07–6.60) | 5.80 (5.30–6.30) | Mann–Whitney U (exact) | 0.5563 |
| ACA RI - before | 0.84 (0.82–0.88) | 0.83 (0.81–0.86) | Mann–Whitney U (exact) | 0.7770 |
| ACA RI - after | 0.83 (0.81–0.87) | 0.79 (0.77–0.81) | Mann–Whitney U (exact) | 0.2943 |
| Celiac PSV - before | 67.80 (53.75–77.25) | 79.00 (77.50–80.50) | Mann–Whitney U (exact) | 0.2575 |
| Celiac PSV - after | 59.00 (51.75–69.58) | 66.50 (63.75–69.25) | Mann–Whitney U (exact) | 0.4138 |
| Celiac EDV - before | 10.00 (7.35–12.00) | 10.40 (10.00–10.80) | Mann–Whitney U (exact) | 0.8368 |
| Celiac EDV - after | 10.30 (7.95–11.25) | 11.50 (10.75–12.25) | Mann–Whitney U (exact) | 0.5563 |
| Celiac RI - before | 0.85 (0.82–0.89) | 0.82 (0.80–0.85) | Mann–Whitney U (exact) | 0.6069 |
| Celiac RI - after | 0.82 (0.81–0.87) | 0.81 (0.81–0.81) | Mann–Whitney U (exact) | 0.4138 |

***Significant p-values (<0.05) are shown in bold red. NEC necrotizing enterocolitis***
